# Guajadials C-F, four unusual meroterpenoids from *Psidium guajava*

**DOI:** 10.1007/s13659-012-0102-4

**Published:** 2013-02-05

**Authors:** Yuan Gao, Gen-Tao Li, Yan Li, Ping Hai, Fei Wang, Ji-Kai Liu

**Affiliations:** 1102 State Key Laboratory of Phytochemistry and Plant Resources in West China, Kunming Institute of Botany, Chinese Academy of Sciences, Kunming, 650201 China; 2102 BioBioPha Co., Ltd., Kunming, 650201 China; 3102 University of Chinese Academy of Sciences, Beijing, 100049 China

**Keywords:** *Psidium guajava*, meroterpenoid, guajadial

## Abstract

**Abstract:**

Guajadials C-F (**1–4**), four sesquiterpenoid-based meroterpenoids with unprecedented skeletons were isolated from the leaves of *Psidium guajava*. Their structures and relative configurations were established by extensive spectroscopic analysis. A possible biosynthetic pathway for **1–4** was also proposed.

**Graphical abstract:**

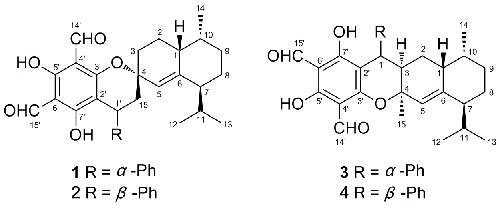

**Electronic Supplementary Material:**

Supplementary material is available for this article at 10.1007/s13659-012-0102-4 and is accessible for authorized users.

## Introduction

*Psidium guajava* L. (Myrtaceae) is an evergreen shrub grown in tropical and subtropical regions as a food, and is also an indigenous medicinal plant used to treat inflammation, diabetes, hypertension, wounds, pain, fever, vomiting, and diarrhea.[1] In recent years, several sesquiterpene-based meroterpenoids with unusual skeletons have been discovered from the leaves of *P. guajava*,[2] some of which exhibited significant biological activities including inhibitory effects on proton tyrosine phosphatase 1B (TPT1B)[2b] and the growth of human hepatoma cell (HepG2 and HepG2/ADM).[2c,d] Plausible biosynthetic pathways[2a,b,d] and biomimetic synthesis[2e,3] relative to *Psidium* meroterpenoids had also stimulated considerable interest in several laboratories. In continuation of our studies on structurally unique and biologically active *Psidium* meroterpenoids, by which two rare meroterpenoids guajadial[2a] and guajadial B[2e] had previously been discovered, another four novel meroterpenoids guajadials C–F (**1**–**4**) were isolated from the leaves of *P. guajava*. Herein, we describe the isolation, structure elucidation, plausible biosynthetic pathway, and cytotoxicity of the new compounds.

**guajadial C (1)**

**guajadial D (2)**

**guajadial E (3)**

**guajadial F (4)**

## Results and Discussion

Guajadial C (**1**), obtained as amorphous powder, $[\alpha]^{24}_{D}$ + 93.5 (*c* 0.20, $CHCl_3$). It possessed a molecular formula of $C_{30}H_{34}O_5$, as evidenced by high-resolution EI mass spectrum at *m*/*z* 474.2401 (calcd 474.2406), in combination with the $^1H$ and $^{13}C$ NMR spectra (Table 1), requiring 14 degrees of unsaturation. The IR spectrum suggested the presence of hydroxyl (3441 $cm^{-1}$) and conjugated carbonyl (1633 $cm^{-1}$) in **1**. The UV spectrum (MeOH) of **1** showed the absorptions maxima at 278 and 337 (sh) nm. Analysis of the NMR data displayed the presence of two chelated phenolic hydroxyls, two aldehydes, three methyls, a monosubstituted benzene ring, and a hexasubstituted aromatic ring (Table 1). These spectroscopic features implied that **1** was a benzyldiformylphloroglucinol-coupled sesquiterpenoid.[2]

In the HMBC spectrum (Figure 1), cross-peaks from H-1′ to C-2′, C-3′, C-7′, C-8′, C-9′, and C-13′ supported the existence of a benzyldiformylphloroglucinol moiety (**1a**). The HSQC and $^1H$-$^1H$ COSY spectra (Figure 1) revealed the connections of C-3/C-2/C-1/C-10/C-9/C-8/C-7/C-11, C-12/C-11/C-13, and C-10/C-14, which were confirmed by HMBC correlations from H-12 to C-11, C-13, and C-7, H-7 to C-8 and C-9, H-14 to C-9, C-10, and C-1, and H-3 to C-1 and C-2. Meanwhile, cross peaks detected from HMBC spectrum, from H-7 to C-1,

*To whom correspondence should be addressed. E-mail: jkliu@mail.kib.ac.cn

**Table 1. $^{1}$H and $^{13}$C NMR data[a] for 1 and 2 in $CDCl_3$**

| | 1 | | 2 | |
|---|---|---|---|---|
| no. | $\delta_H$ ($J$ in Hz) | $\delta_C$ | $\delta_H$ ($J$ in Hz) | $\delta_C$ |
| 1 | 2.27 (m) | 37.6 | 2.25 (m) | 37.3 |
| 2$\alpha$ | 1.65 (m) | 21.9 | 1.79 (m) | 22.0 |
| 2$\beta$ | 1.56 (m) | | 1.56 (m) | |
| 3$\alpha$ | 2.07 (m) | 30.8 | 1.90 (ddd, 13.2, 5.6, 4.0) | 34.3 |
| 3$\beta$ | 1.45 (td, 13.3, 3.4) | | 1.34 (m) | |
| 4 | | 78.0 | | 77.9 |
| 5 | 5.33 (s) | 125.4 | 5.59 (s) | 123.0 |
| 6 | | 147.9 | | 148.2 |
| 7 | 1.57 (m) | 50.6 | 1.62 (m) | 50.9 |
| 8$\alpha$ | 1.68 (m) | 22.1 | 1.64 (m) | 22.2 |
| 8$\beta$ | 1.65 (m) | | 1.63 (m) | |
| 9$\alpha$ | 1.39 (br. d, 13.4) | 28.7 | 1.36 (br. d, 13.2) | 28.7 |
| 9$\beta$ | 1.81 (m) | | 1.80 (m) | |
| 10 | 1.98 (m) | 33.0 | 1.97 (m) | 33.5 |
| 11 | 1.76 (m) | 26.9 | 1.82 (m) | 26.9 |
| 12 | 0.57 (d, 6.6) | 21.5 | 0.75 (d, 6.6) | 21.6 |
| 13 | 0.88 (d, 6.5) | 21.2 | 0.93 (d, 6.5) | 21.3 |
| 14 | 0.90 (d, 6.9) | 14.3 | 0.87 (d, 7.0) | 14.3 |
| 15$\alpha$ | 2.04 (dd, 14.3, 10.5) | 43.7 | 2.25 (dd, 14.2, 7.3) | 43.2 |
| 15$\beta$ | 2.16 (dd, 14.3, 7.1) | | 2.02 (dd, 14.2, 9.3) | |
| 1′ | 4.06 (dd, 10.5, 7.1) | 34.6 | 4.19 (dd, 9.3, 7.3) | 34.1 |
| 2′ | | 102.8 | | 102.9 |
| 3′ | | 164.6 | | 164.7 |
| 4′ | | 104.5 | | 104.6 |
| 5′ | | 168.6 | | 168.6 |
| 6′ | | 104.0 | | 104.0 |
| 7′ | | 169.8 | | 169.7 |
| 8′ | | 144.7 | | 144.5 |
| 9′, 13′ | 7.15 (d, 7.3) | 126.7 | 7.15 (d, 7.2) | 126.8 |
| 10′, 12′ | 7.29 (t, 7.5) | 128.5 | 7.28 (t, 7.6) | 128.5 |
| 11′ | 7.21 (t, 7.4) | 126.3 | 7.21 (t, 7.3) | 126.2 |
| 14′ | 10.10 (s) | 192.4 | 10.10 (s) | 192.3 |
| 15′ | 10.12 (s) | 191.6 | 10.13 (s) | 191.6 |
| 5′-OH | 13.58 (s) | | 13.57 (s) | |
| 7′-OH | 13.17 (s) | | 13.21 (s) | |

[a]Assignments based on HMBC, HSQC, $^{1}$H-$^{1}$H COSY, and ROESY spectra.

C-5, and C-6 and H-15 to C-3, C-4, and C-5, revealed the C-linkages of C-3/C-4/C-5/C-6/C-7, C-1/C-6, and C-15/C-4. The above evidence strongly suggested that compound **1** possessed a sesquiterpenoid moiety (**1b**) resembled scapanol.[4] Comparison of the $^{1}$H and $^{13}$C NMR data assigned to **1b** with those of scapanol indicated that they were similar, except that Me-15 of scapanol was replaced by a methylene and a significant downfield shift ($\Delta \approx 10.3$ ppm) for C-4 of **1b** was observed. The above observation implied that **1b** was fused to **1a** via C-4 and C-15, which was further confirmed by the HMBC correlations from H-1′ to C-4 and C-15, as well as the degrees of unsaturation requiring the existence of an ether bridge between C-4 and C-3′.

**Figure 1.** Key $^{1}$H-$^{1}$H COSY and HMBC correlations of **1** and **3**

The relative configuration of **1** was established by analysis of the ROESY data and proton coupling constants based on computer-generated 3D drawing with minimized energy by MM2 calculation using ChemBio3D software. In the ROESY spectrum, the *pseudo*-axially oriented proton H-3$\beta$ at $\delta_H$ 1.45 (td, 13.3 and 3.4 Hz) in the half-chair cyclohexene ring B showed correlations with H-1 and Me-12, revealing the H-1 and isopropyl group to be $\beta$- and axially oriented. In addition, correlations between Me-14 and H-2$\alpha$/H-8$\alpha$ suggested the Me-14 adopts another orientation. This deduction was further supported by the narrow half-peak width of H-10 (19.3 Hz), which suggested that in the chair cyclohexane ring A, the H-10 was $\beta$- and equatorially oriented and accordingly the Me-14 was $\alpha$- and axially oriented. The correlations among H-15$\beta$, Me-12, and H-3$\beta$ indicated the $\beta$-orientation of the methylene at C-4. The $\alpha$-orientation of the phenyl was strongly suggested by the significant correlation of H-1′↔H-3$\alpha$, as also supported by the large coupling constant ($J_{1'\text{-}15\alpha}$ = 10.5 Hz) which indicated a *trans pseudo*-diaxial relationship for H-1′ and H-15$\alpha$ in the half-chair dihydropyran ring. Therefore, these analyses established the relative configuration and the conformation of the A–B–C ring to be chair–half-chair–half-chair, as shown in Figure 2.

Guajadial D (**2**) was isolated as amorphous powder, $[\alpha]_D^{24}$ + 45.4 ($c$ 0.20, $CHCl_3$). Its molecular formula of $C_{30}H_{34}O_5$ can be deduced by HREIMS spectrum, $[M]^+$ peak at $m/z$ 474.2410 (calcd 474.2406). The UV spectrum (MeOH) showed absorptions at $\lambda_{max}$ 278 and 337 (sh) nm. The NMR spectra also displayed resonances for benzyldiformylphloroglucinol and scapanol moieties (Table 1). The HMBC correlations from H-7 to C-1/C-5/C-6, Me-14 to C-1/C-9/C-10, and H-15 to C-3/C-4/C-5, and comparison with $^{1}$H and $^{13}$C NMR spectroscopic data of **1**, are in agreement with a meroterpenoid possessing the same planar structure as **1**. Detailed analysis of its ROESY correlations (Figure 2), among which H-1↔H-11, Me-14↔H-2$\alpha$/H-8$\alpha$, and H-15$\alpha$↔Me-12/H-5 established the relative configuration of the sesquiterpenoid unit identical to that of **1**, however, revealed as a major difference from **1**, the discrepant orientation of phenyl. The significant ROESY correlations between H-1′ and H-3$\alpha$ fixing the phenyl of **1** as $\alpha$-oriented was no longer observable in the ROESY spectrum of **2**, instead, strong correlation between H-1′ and H-5 was observed, indicating the $\beta$-orientation of phenyl. Consequently, the relative configuration of **2** was determined as shown in Figure 2.

Guajadial E (**3**) was obtained as amorphous powder, $[\alpha]_D^{24}$ +

**Figure 2.** Key ROESY correlations of **1**–**4**

91.1 ($c$ 0.20, $CHCl_3$). The molecular formula was assigned as $C_{30}H_{34}O_5$ by HREIMS at $m/z$ 474.2399 $[M]^+$ (calcd 474.2406). The UV spectrum (MeOH) showed absorptions at $\lambda_{max}$ 277 and 337 (sh) nm. The NMR spectra (Table 2) also displayed signals for partial structure of benzyldiformylphloroglucinol (**3a**) besides which there were signals for four methyls, three methylenes, five methines, one oxygenated quarternary carbon, and a trisubstituted double bond that could be ascribed to a sesquiterpenoid unit with the aid of HSQC, $^1$H-$^1$H COSY, and HMBC experiments (Figure 1). The HSQC and $^1$H-$^1$H COSY spectra revealed the connections of C-1′/C-3/C-2/C-1/C-10/C-9/C-8/C-7/C-11, C-12/C-11/C-13, and C-10/C-14. In the HMBC spectrum, cross peaks from H-7 to C-1, C-5, and C-6 and Me-15 to C-3, C-4, and C-5 established the C-linkage of C-3/C-4/C-5/C-6/C-7, C-1/C-6, and C-15/C-4. The above evidence allowed the construction of a basic structural unit of a sesquiterpenoid **3b**, to which signals assigned were analogous to those of **1b**. Different from **1b**, the C-3 methylene was replaced by a methine, meanwhile, the C-15 methylene was changed into a methyl group, suggesting a dihydropyran ring was fused to **3b** via C-3 and C-4 instead of C-4 and C-15. The linkage between fragments **3a** and **3b** was further supported by HMBC experiment and molecular formula information. In the HMBC spectrum, correlations of H-1′ with C-2, C-3, and C-4 revealed the connection of C-1′/C-3. According to the molecular formula information, the oxygen atom leftover was to bridge C-4 and C-3′, to form a dihydropyran ring.

The relative configuration for **3** could be deduced by its ROESY spectrum. The ROESY correlations of H-1↔H-11, Me-14↔H-2$\alpha$/H-8$\alpha$, Me-15↔H-3, and H-1′↔H-1/Me-12/H-2$\beta$ established the relative configuration of **3** as shown in Figure 2.

Guajadial F (**4**) was obtained as amorphous powder, $[\alpha]_D^{24}$ + 191.3 ($c$ 0.21, $CHCl_3$). The $[M]^+$ at m/z 474.2410 (calcd 474.2406) in its HREIMS gave a molecular formula $C_{30}H_{34}O_5$. The UV spectrum (MeOH) showed absorptions at $\lambda_{max}$ 278 and 342 nm. Careful comparison of its $^1$H and $^{13}$C NMR data (Table 2) with those of **3** suggested that they also share the same planar structure, which was further confirmed by HMBC

**Table 2.** $^1$H and $^{13}$C NMR data[a] for 3 and 4 in $CDCl_3$

| no. | 3: $\delta_H$ ($J$ in Hz) | 3: $\delta_C$ | 4: $\delta_H$ ($J$ in Hz) | 4: $\delta_C$ |
|---|---|---|---|---|
| 1 | 2.63 (m) | 34.1 | 2.30 (br. t, 6.7) | 35.1 |
| 2$\alpha$ | 1.65 (m) | 27.0 | 0.58 (dd, 14.3, 3.9) | 25.9 |
| 2$\beta$ | 1.77 (m) | | 1.43 (ddd, 14.3, 14.3, 8.4) | |
| 3 | 2.19 (m) | 44.4 | 2.15 (ddd, 14.3, 7.1, 3.9) | 38.1 |
| 4 | | 80.0 | | 78.0 |
| 5 | 5.31 (s) | 126.7 | 5.59 (d, 1.4) | 125.7 |
| 6 | | 144.5 | | 147.2 |
| 7 | 1.58 (m) | 50.8 | 1.72 (m) | 51.0 |
| 8$\alpha$ | 1.63 (m) | 22.6 | 1.56 (m) | 22.8 |
| 8$\beta$ | 1.68 (m) | | 1.65 (m) | |
| 9$\alpha$ | 1.40 (br. d, 13.4) | 29.0 | 1.16 (br. d, 13.6) | 28.8 |
| 9$\beta$ | 1.85 (m) | | 1.73 (m) | |
| 10 | 2.00 (m) | 33.7 | 1.64 (m) | 36.7 |
| 11 | 1.82 (m) | 26.4 | 1.81 (m) | 26.3 |
| 12 | 0.52 (d, 6.5) | 21.1 | 0.78 (d, 6.5) | 21.4 |
| 13 | 0.87 (d, 6.5) | 21.1 | 0.91 (d, 6.4) | 21.0 |
| 14 | 0.92 (d, 7.0) | 14.3 | 0.65 (d, 7.1) | 14.8 |
| 15 | 1.45 (s) | 28.1 | 1.50 (s) | 24.3 |
| 1′ | 3.67 (d, 6.9) | 38.6 | 4.41 (d, 7.1) | 38.2 |
| 2′ | | 104.3 | | 100.5 |
| 3′ | | 163.4 | | 164.2 |
| 4′ | | 104.0 | | 104.4 |
| 5′ | | 168.2 | | 168.5 |
| 6′ | | 104.0 | | 104.1 |
| 7′ | | 169.7 | | 170.3 |
| 8′ | | 144.5 | | 140.7 |
| 9′ | 7.14 (d, 7.2) | 127.6 | 6.81 (m) | 127.6 |
| 10′ | 7.27 (t, 7.5) | 128.3 | 6.70–7.45 (m) | 127.6[b] |
| 11′ | 7.20 (t, 7.3) | 126.2 | 7.23 (t, 6.9) | 126.2 |
| 12′ | 7.27 (t, 7.5) | 128.3 | 6.70–7.45 (m) | 128.5[b] |
| 13′ | 7.14 (d, 7.2) | 127.6 | 6.70–7.45 (m) | 127.6[b] |
| 14′ | 10.14 (s) | 192.4 | 10.02 (s) | 192.5 |
| 15′ | 10.09 (s) | 191.6 | 10.13 (s) | 191.5 |
| 5′-OH | 13.50 (s) | | 13.51 (s) | |
| 7′-OH | 13.05 (s) | | 13.31 (s) | |

[a]Assignments based on HMBC, HSQC, $^1$H–$^1$H COSY, and ROESY spectra. [b]Interchangable signals.

correlations from H-7 to C-1/C-5/C-6, Me-15 to C-3/C-4/C-5, Me-14 to C-9/C-10/C-1, and H-1′ to C-2/C-3/C-3′/C-7′/C-9′/C-13′. In the ROESY spectrum (Figure 2), correlations of H-1↔H-11, Me-14↔H-3/8$\alpha$, and Me-15↔H-3 established the relative configuration of the sesquiterpenoid moiety which was identical to that of **3**. Meanwhile, the correlation of H-1′↔Me-15 implied that **4** was a C-1′ epimer of **3**. The noticeable difference between the chemical shift value of H-2$\alpha$ (**3**: $\delta_H$ 1.65; **4**: $\delta_H$ 0.58) also provided evidence that the phenyl had a different orientation because the significant up field shift ($\Delta \approx$ 1.07 ppm) can be explained by the anisotropic effect of phenyl

**Scheme 1.** Plausible biosynthetic pathway for **1**–**4**

as shown in the computer-generated 3D drawing. Thus, the relative configuration of **4** was elucidated as shown in Figure 2.

Because the key intermediate 3,5-dimethyl-2,4,6-trihydroxy-benzophenon had been previously isolated from the same plant,[5] the plausible biogenetic route of **1**–**4** could be proposed as shown in Scheme 1. The intermediate could be oxidized and then generated carbocation **A**, then **A** could attack **5** and **6** to generate tertiary carbocations **B**–**E** with resonance stabilization. Further cyclization of **B**–**E** could construct compounds **1**–**4**.

Compounds **1**–**4** were evaluated for their *in vitro* growth inhibitory effects against five human cancer cell lines (MCF-7, A-549, SMMC-7721, SW480, and HL-60). All of the compounds exhibited positive activity ($IC_{50}$ 5.59–40.0 $\mu$M) toward all five human cancer cell lines except that compound **1** was inactive to MCF-7 (Table 3).

**Table 3. Cytotoxicity data of compounds 1–4 with $IC_{50}$ values ($\mu$M)**

| compound | Cell line | | | | |
|---|---|---|---|---|---|
| | MCF-7 | A-549 | SMMC-7721 | SW480 | HL-60 |
| **1** | > 40.00 | 24.96 | 19.37 | 27.65 | 19.39 |
| **2** | 40.00 | 38.08 | 27.47 | 34.41 | 22.51 |
| **3** | 7.78 | 6.30 | 5.59 | 13.39 | 7.77 |
| **4** | 16.59 | 14.16 | 13.31 | 15.10 | 12.84 |
| cisplatin[a] | 13.42 | 8.55 | 5.58 | 12.10 | 1.24 |

[a]Cisplatin was used as positive control.

## Experimental Section

**General Experimental Procedures.** Optical rotations were measured on a Jasco P-1020 automatic digital polarimeter. IR spectra were obtained using a Bruker Tensor 27 FT-IR spectrometer with KBr pellets. NMR spectra were acquired with an Avance III 600 instrument at room temperature. EIMS (including HREIMS) were measured on VG-Auto-Spec-3000 spectrometers. Silica gel (200–300 mesh, Qingdao Marine Chemical Inc., China) and Sephadex LH-20 (Amersham Biosciences, Sweden) were used for column chromatography. Fractions were monitored by TLC (Qingdao Marine Chemical

Inc., China) in combination with reversed-phase HPLC (Agilent 1100, Extend-C18 column, 5 $\mu$m, 4.6 × 150 mm). Prep. HPLC was performed using an Agilent 1100 series (ZORBAX SB-C18 column, 5 $\mu$m, 21.2 × 150 mm for 20 mL/min). Silica gel for prep. TLC was obtained from Qingdao Marine Chemical Inc., China.

**Plant Material.** Leaves of *P. guajava* were collected from south of Vietnam in 2009 and were identified by Prof. Yu Chen of Kunming Institute of Botany, Chinese Academy of Sciences. The voucher specimen was deposited at BioBioPha Co., Ltd.

**Extraction and Isolation.** Leaves of *P. guajava* (15 kg) were extracted with MeOH at room temperature. After filtration, the methanolic extract was evaporated under reduced pressure to get a residue (*ca.* 1400 g), which was fractionized by silica gel column chromatography using petroleum ether containing an increasing amount of acetone. Seperation of 2.5 g of the fraction eluted with petroleum ether–acetone (200:1) by Sephadex LH-20 ($CHCl_3$–MeOH, 1:1) and prep. HPLC on a ZORBAX SB-C18 column (84% $CH_3CN$ in $H_2O$ and 0.1% formic acid over 17 min followed by 84%→100% $CH_3CN$ in $H_2O$ and 0.1% formic acid to 24 min, 20 mL/min, 280 nm) yielded four fractions A–D. Fraction C (22 mg, $t_R$ = 13.5→14.5 min) was further purified by prep. TLC (petroleum ether/EtOAc/formic acid, 50:2:0.1) to give **3** (7 mg, $R_f \approx 0.6$). Fraction D (50 mg, $t_R$ = 17.1→19.1 min) was purified further by prep. HPLC on a ZORBAX SB-C18 column (90%→92% $CH_3CN$ in $H_2O$ and 0.1% formic acid over 12 min followed by 92%→100% $CH_3CN$ in $H_2O$ and 0.1% formic acid to 15 min, 20 mL/min, 280 nm) and then prep. TLC (petroleum ether/EtOAc/formic acid, 50:2:0.1) to afford **1** (8 mg, $t_R$ = 12.5 min, $R_f \approx 0.5$), **2** (8 mg, $t_R$ = 12.5 min, $R_f \approx 0.6$), and **4** (7 mg, $t_R$ = 10.2 min, $R_f \approx 0.5$).

**Guajadial C (1):** amorphous powder; $[\alpha]_D^{24}$ + 93.5 (*c* 0.20, $CHCl_3$); UV (MeOH) $\lambda_{max}$: 278, 337 (sh) nm; IR (KBr) $\nu_{max}$ 3441, 2956, 2926, 2870, 1633, 1440, 1383, 1302, 1266, 1230, 1211, 1059, 848, 829 $cm^{-1}$; $^1$H and $^{13}$C NMR data see Table 1; EIMS: *m/z* 474 $[M]^+$ (100), 431 (64), 327 (23), 283 (20), 271 (27), 195 (22), 161 (32), 119 (10), 105 (14), 91 (16); HREIMS: *m/z* 474.2401 (calcd 474.2406).

**Guajadial D (2):** amorphous powder; $[\alpha]_D^{24}$ + 45.4 (*c* 0.20, $CHCl_3$); UV (MeOH) $\lambda_{max}$: 278, 337 (sh) nm; IR (KBr) $\nu_{max}$ 3442, 2956, 2927, 2870, 1634, 1440, 1383, 1367, 1302, 1183, 1156, 1125, 1098, 849, 762 $cm^{-1}$; $^1$H and $^{13}$C NMR data see Table 1; EIMS: *m/z* 474 $[M]^+$ (100), 431 (61), 327 (29), 283 (20), 271 (24), 195 (11), 161 (12), 119 (4), 105 (6), 91 (5); HREIMS: *m/z* 474.2410 (calcd 474.2406).

**Guajadial E (3):** amorphous powder; $[\alpha]_D^{24}$ + 91.1 (*c* 0.20, $CHCl_3$); UV (MeOH) $\lambda_{max}$: 277, 337 (sh) nm; IR (KBr) $\nu_{max}$ 3440, 2958, 2927, 2872, 1632, 1439, 1383, 1305, 1194, 1180, 1158, 1144, 1077, 699, 608 $cm^{-1}$; $^1$H and $^{13}$C NMR data see Table 2; EIMS: *m/z* 474 $[M]^+$ (71), 431 (18), 292 (60), 272 (99), 244 (55), 204 (60), 161 (100), 119 (69), 105 (66), 91 (31), 81 (26), 69 (15), 55 (20); HREIMS: *m/z* 474.2399 (calcd 474.2406).

**Guajadial F (4):** amorphous powder; $[\alpha]_D^{24}$ + 191.3 (*c* 0.21, $CHCl_3$); UV (MeOH) $\lambda_{max}$: 278, 342 nm; IR (KBr) $\nu_{max}$ 3424, 2957, 2930, 2869, 1635, 1439, 1382, 1301, 1266, 1238, 1187, 1154, 1134, 1090, 846, 775 $cm^{-1}$; $^1$H and $^{13}$C NMR data see Table 2; EIMS: *m/z* 474 $[M]^+$ (25), 431 (11), 272 (100), 244 (50), 204 (54), 161 (85), 119 (81), 105 (68), 91 (49), 81 (28), 69 (14), 55 (10); HREIMS: *m/z* 474.2410 (calcd 474.2406).

**Cytotoxicity Bioassays.** The following human tumor cell lines were used: HL-60, SMMC-7721, A-549, MCF-7, and SW480. All cells were cultured in RPMI-1640 or DMEM medium (Hyclone, Logan, UT), supplemented with 10% fetal bovine serum (Hyclone) at 37 °C in a humidified atmosphere with 5% $CO_2$. The cytotoxicity assay was performed according to an MTS (3-(4,5-dimethylthiazol-2-yl)-5-(3-carboxymethoxy-phenyl)-2-(4-sulfopheny)-2H-tetrazolium) method[6] with minor modifications. Briefly, 100 $\mu$L of adherent cells were seeded into each well of a 96-well cell culture plate and allowed to adhere for 12 h before drug addition, while suspended cells were seeded just before drug addition, both with an initial density of 1 × $10^5$ cells/mL in 100 $\mu$L medium. Each cell line was exposed to the test compound at various concentrations in triplicate for 48 h, with cisplatin (Sigma) as positive control. After the incubation, 20 $\mu$L MTS and 100 $\mu$L medium was added to each well after removal of 100 $\mu$L medium, and the incubation continued for 4 h at 37 °C. The optical density of the lysate was measured at 490 nm in a 96-well microtiter plate reader (Bio-Rad 680). The $IC_{50}$ value of each compound was calculated by the Reed and Muench's method.[7]

## Electronic Supplementary Material

Supplementary material is available in the online version of this article at http://dx.doi.org/ 10.1007/s13659-012-0102-4 and is accessible for authorized users.

## Acknowledgments

The authors acknowledge the National Basic Research Program of China (973 Program, 2009CB522300), the "West Light" program of Chinese Academy of Sciences, and the National Natural Science Foundation of China (U1132607).

**Open Access** This article is distributed under the terms of the Creative Commons Attribution License which permits any use, distribution, and reproduction in any medium, provided the original author(s) and source are credited.

## Electronic supplementary material

Supplementary material, approximately 2.05 MB.
