# Supplementary material for: Guajadials C-F, four unusual meroterpenoids from Psidium guajava
Source: Nat Prod Bioprospect. 2013 Feb 5;3(1):14–9. doi: 10.1007/s13659-012-0102-4 (PMC4131611; doi:10.1007/s13659-012-0102-4)
Supplement: Supplementary file 1 — Supplementary material, approximately 2.05 MB. [file 13659_2012_102_MOESM1_ESM.pdf]

## Electronic Supplementary Material

# Guajadials C–F, four unusual meroterpenoids from *Psidium guajava*

Yuan GAO,[a,b,c] Gen-Tao LI,[a] Yan LI,[a] Ping HAI,[b] Fei WANG,[a,b] and Ji-Kai LIU[a,]*

[a]State Key Laboratory of Phytochemistry and Plant Resources in West China, Kunming Institute of Botany, Chinese Academy of Sciences, Kunming 650201, China

[b]BioBioPha Co., Ltd., Kunming 650201, China

[c]University of Chinese Academy of Sciences, Beijing 100049, China

Received 23 December 2012; Accepted 29 January 2013

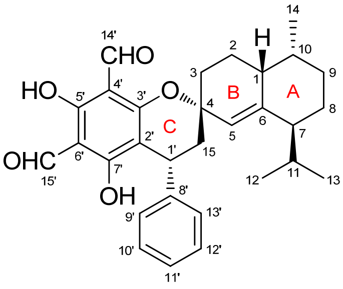


**guajadial C (1)**

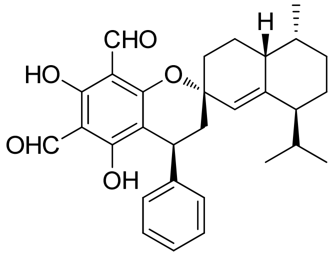


**guajadial D (2)**

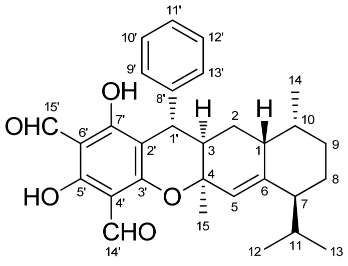


**guajadial E (3)**

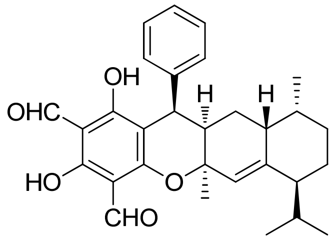


**guajadial F (4)**

Structures of compounds **1**–**4**

*To whom correspondence should be addressed. E-mail: jkliu@mail.kib.ac.cn

## Content list

S1. Plausible biosynthetic pathway for **1–4** (Scheme 1)

S2. $^{1}$H NMR (600 MHz) for guajadial C (**1**)

S3. $^{13}$C NMR (DEPT 150 MHz) for guajadial C (**1**)

S4. HSQC (600 MHz) for guajadial C (**1**)

S5. HMBC (600 MHz) for guajadial C (**1**)

S6. $^{1}$H−$^{1}$H COSY (600 MHz) for guajadial C (**1**)

S7. ROESY (600 MHz) for guajadial C (**1**)

S8. EI-MS for guajadial C (**1**)

S9. HR-EI-MS for guajadial C (**1**)

S10. IR for guajadial C (**1**)

S11. $^{1}$H NMR (600MHz) for guajadial D (**2**)

S12. $^{13}$C NMR (DEPT 150 MHz) for guajadial D (**2**)

S13. HSQC (600 MHz) for guajadial D (**2**)

S14. HMBC (600 MHz) for guajadial D (**2**)

S15. $^{1}$H−$^{1}$H COSY (600 MHz) for guajadial D (**2**)

S16. ROESY (600 MHz) for guajadial D (**2**)

S17. EI-MS for guajadial D (**2**)

S18. HR-EI-MS for guajadial D (**2**)

S19. IR for guajadial D (**2**)

S20. $^{1}$H NMR (600MHz) for guajadial E (**3**)

S21. $^{13}$C NMR (DEPT 150 MHz) for guajadial E (**3**)

S22. HSQC (600 MHz) for guajadial E (**3**)

S23. HMBC (600 MHz) for guajadial E (**3**)

S24. $^{1}$H−$^{1}$H COSY (600 MHz) for guajadial E (**3**)

S25. ROESY (600 MHz) for guajadial E (**3**)

S26. EI-MS for guajadial E (**3**)

S27. HR-EI-MS for guajadial E (**3**)

S28. IR for guajadial E (**3**)

S29. $^{1}$H NMR (600MHz) for guajadial F (**4**)

S30. $^{13}$C NMR (DEPT 150 MHz) for guajadial F (**4**)

S31. The relative areas of peaks of overlapping carbon signals for guajadial F (**4**)

S32. HSQC (600 MHz) for guajadial F (**4**)

S33. HMBC (600 MHz) for guajadial F (**4**)

S34. $^{1}H-^{1}H$ COSY (600 MHz) for guajadial F (**4**)

S35. ROESY (600 MHz) for guajadial F (**4**)

S36. EI-MS for guajadial F (**4**)

S37. HR-EI-MS for guajadial F (**4**)

S38. IR for guajadial F (**4**)

S1. Plausible biosynthetic pathway for **1–4** (Scheme 1)

**Scheme 1.** Plausible Biosynthetic Pathway for **1–4**

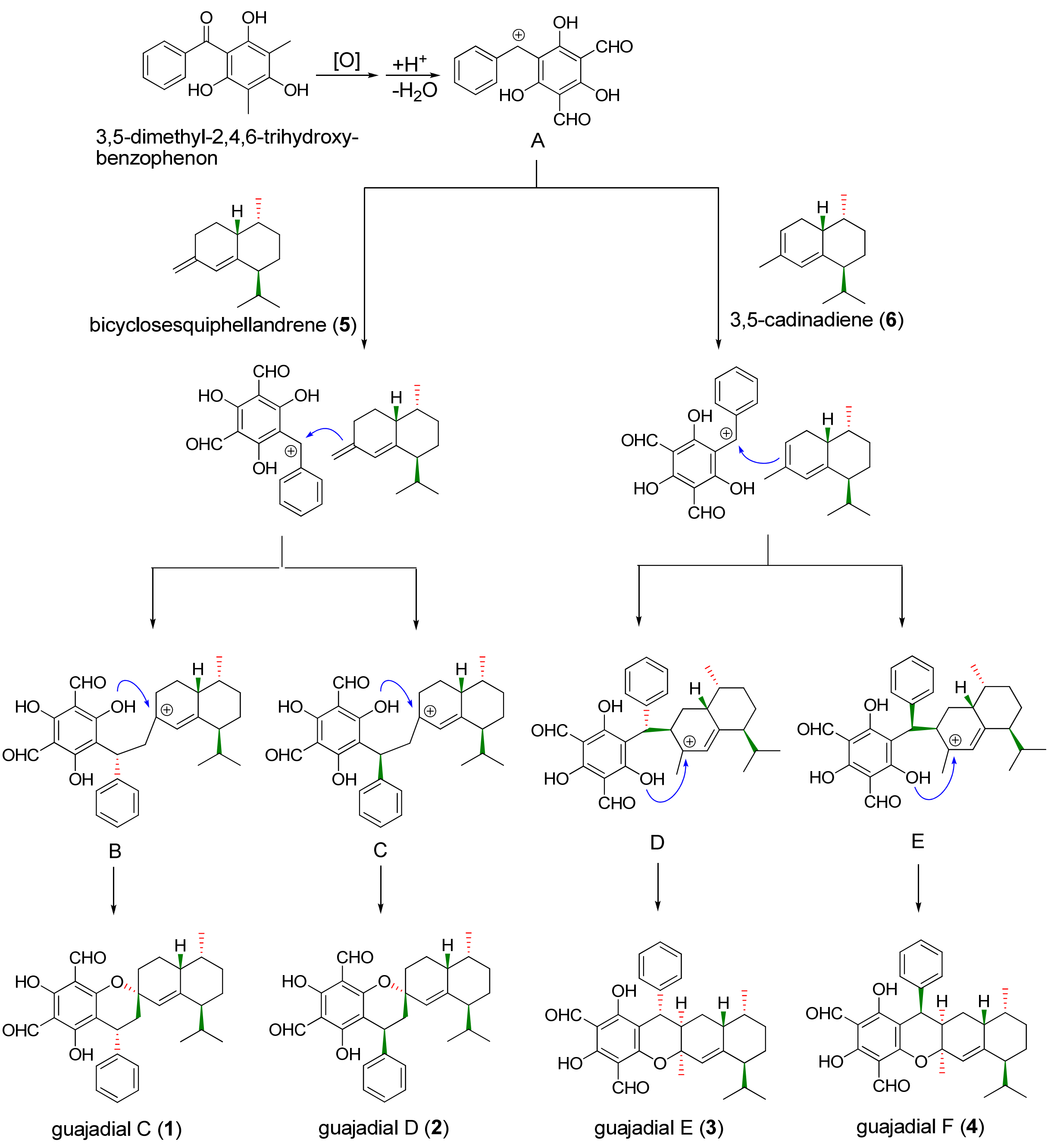

S2. $^{1}$H NMR (600 MHz) for guajadial C (**1**)

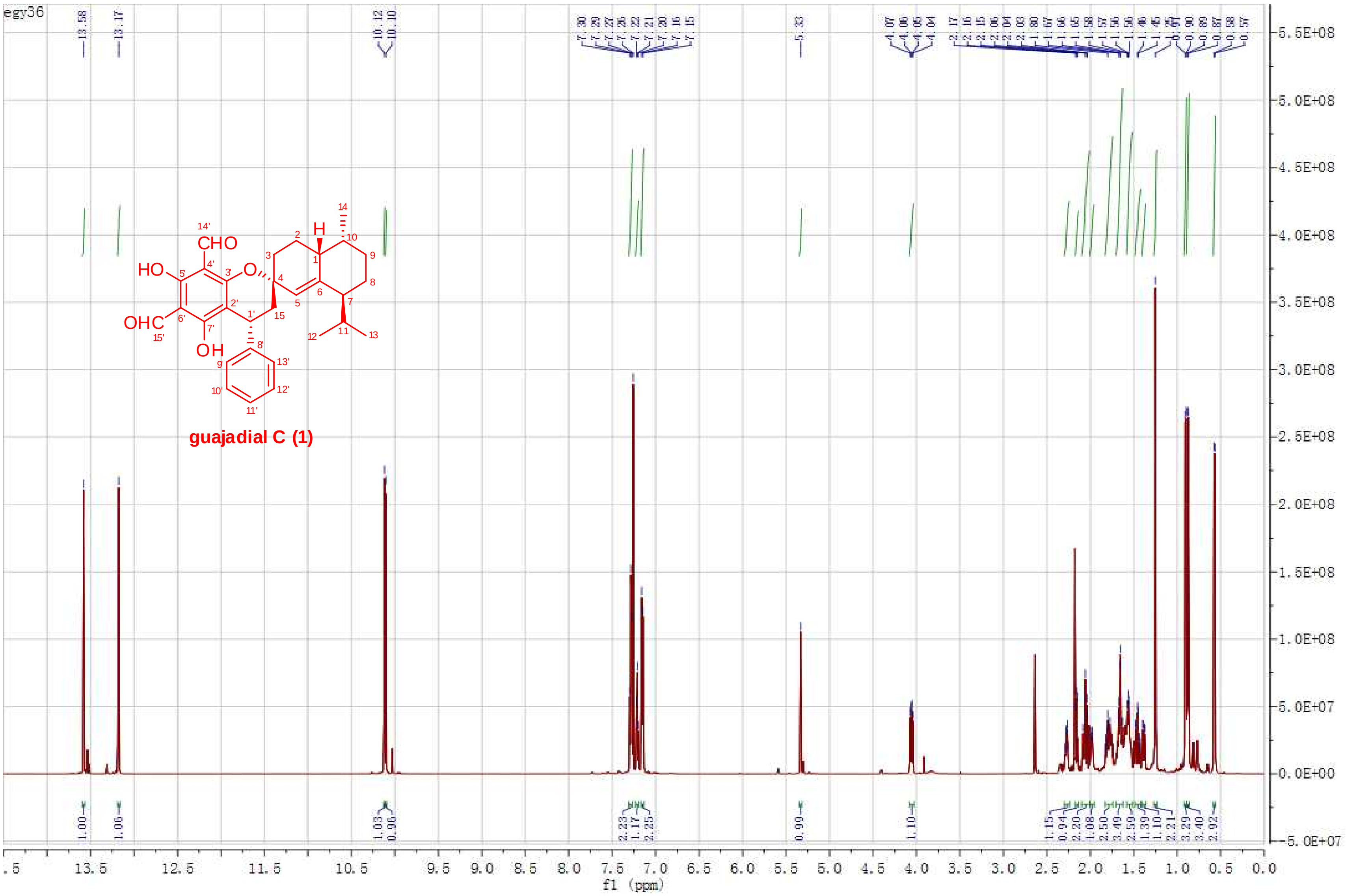

S3. $^{13}$C NMR (DEPT 150 MHz) for guajadial C (**1**)

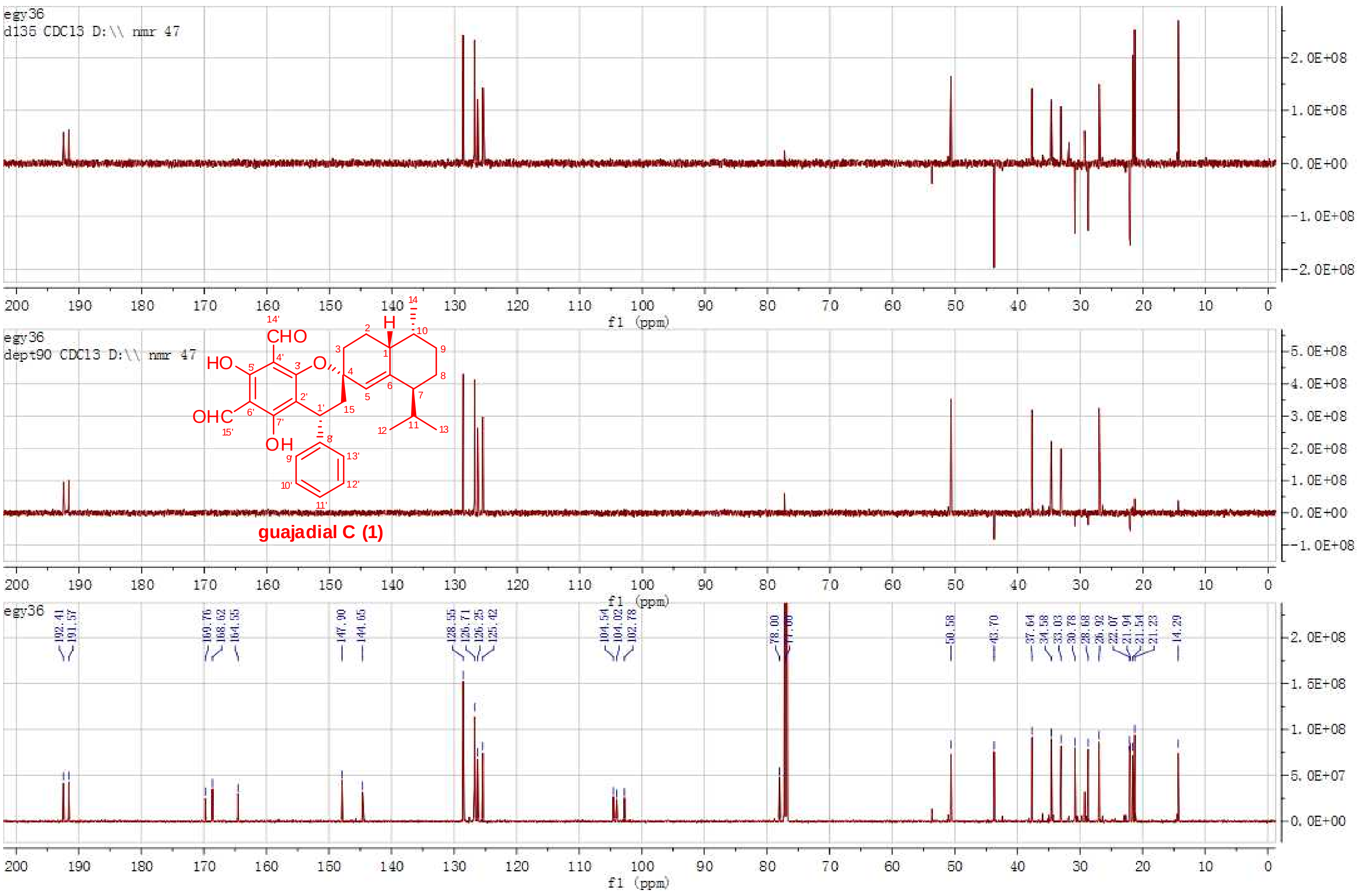

S4. HSQC (600 MHz) for guajadial C (**1**)

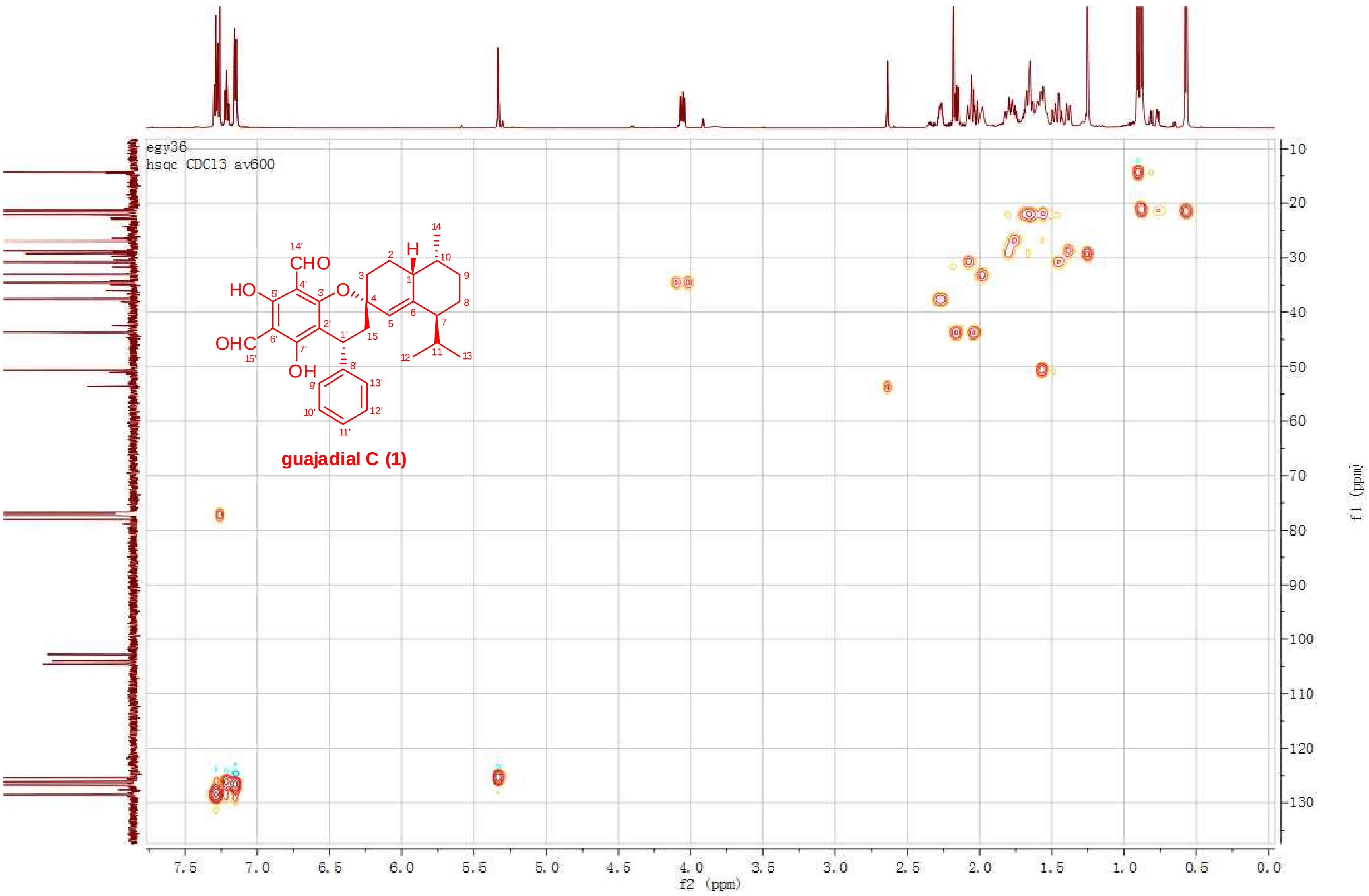

S5. HMBC (600 MHz) for guajadial C (**1**)

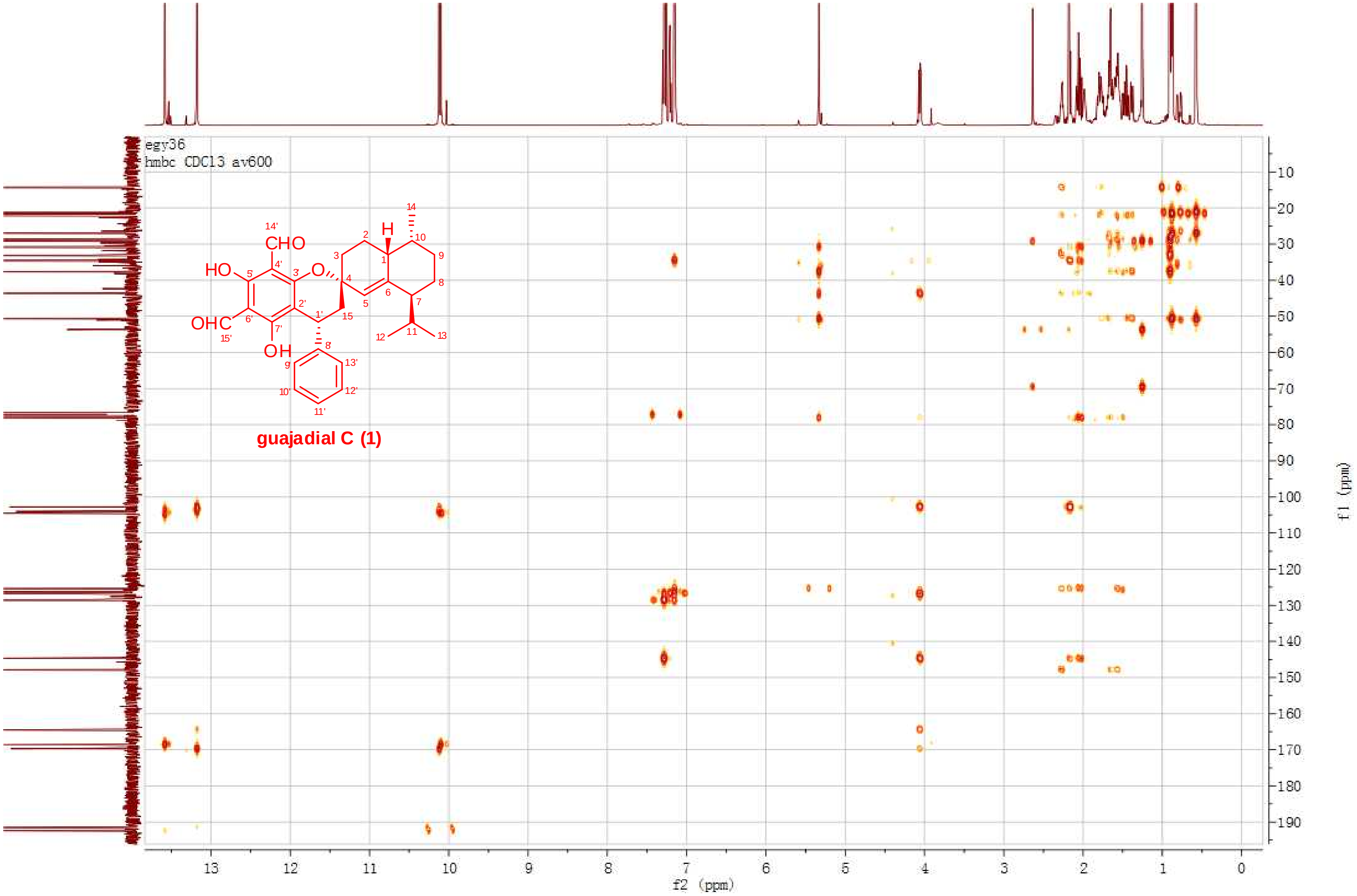

S6. $^{1}H$–$^{1}H$ COSY (600 MHz) for guajadial C (**1**)

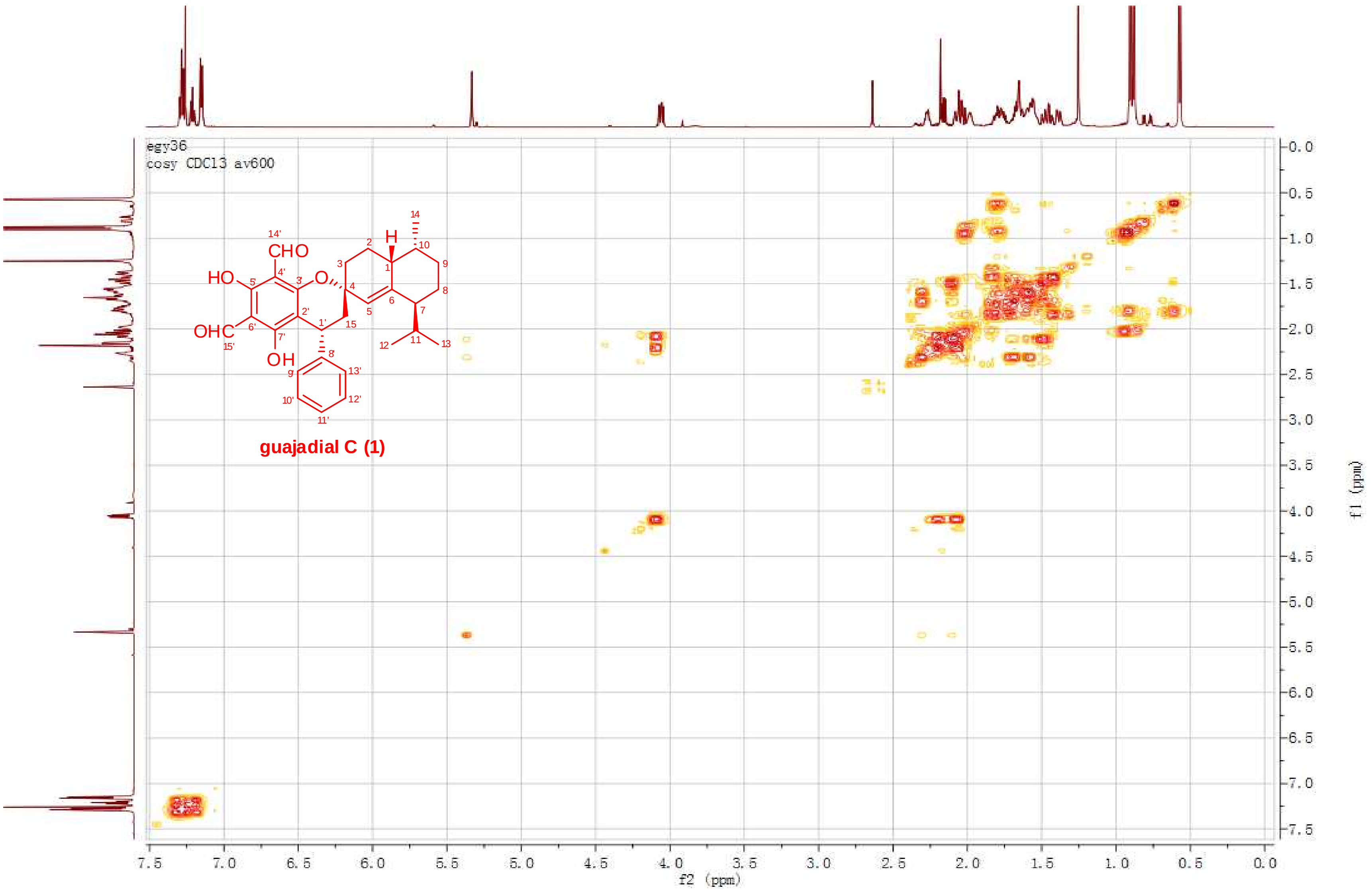

S7. ROESY (600 MHz) for guajadial C (**1**)

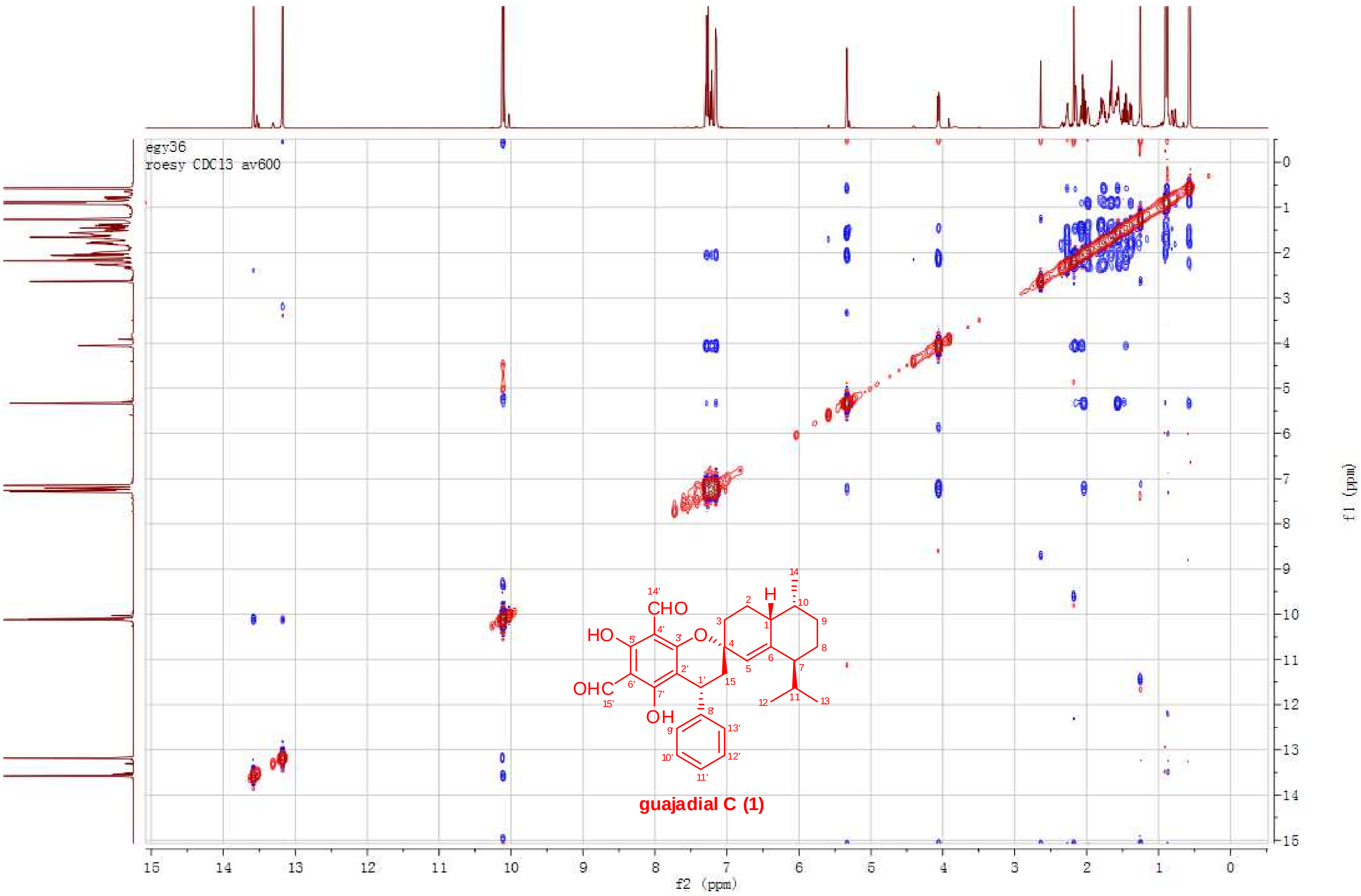

S8. EI-MS for guajadial C (**1**)

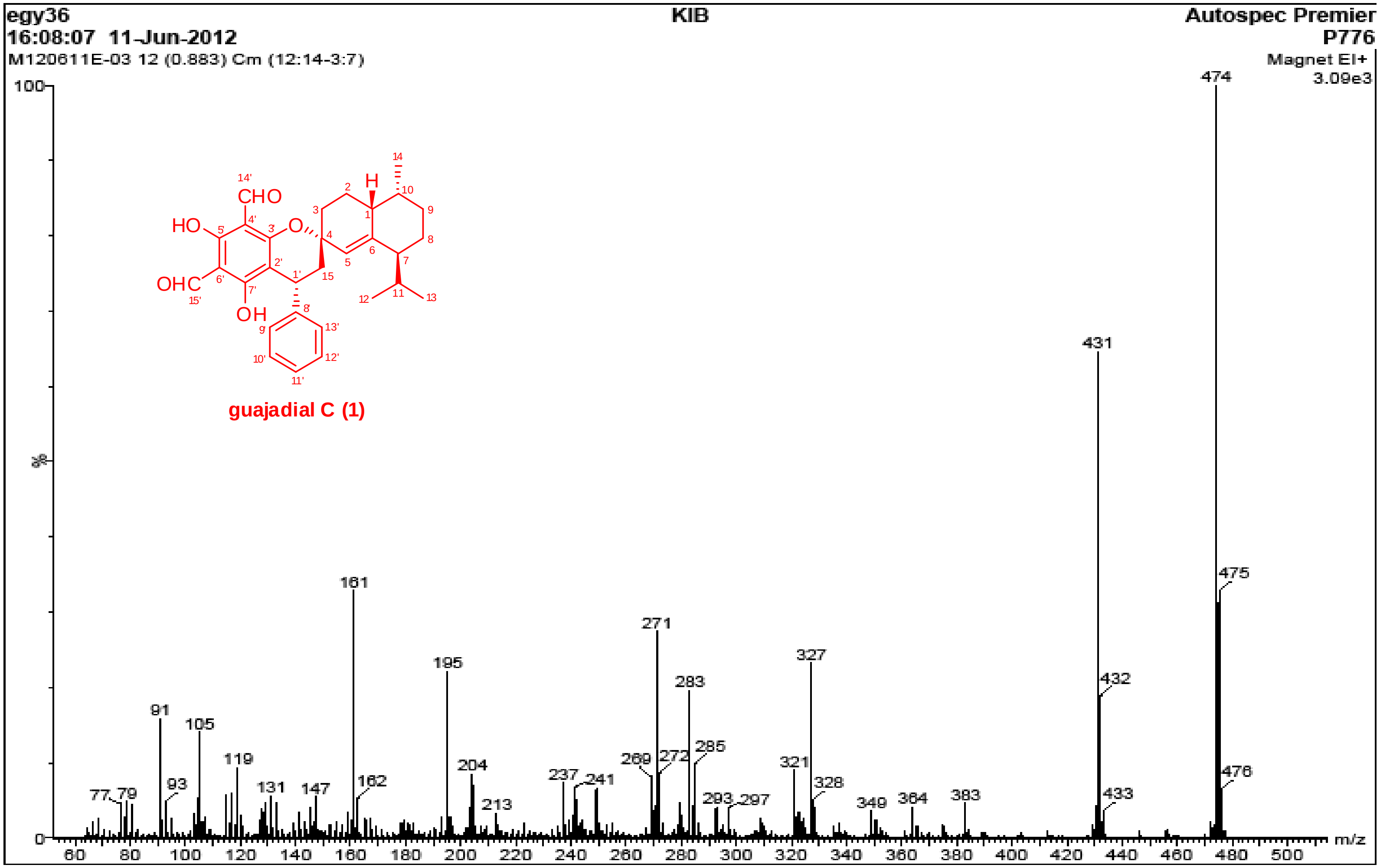

S9. HR-EI-MS for guajadial C (**1**)

## Elemental Composition Report

Page 1

**Single Mass Analysis**
Tolerance = 10.0 PPM / DBE: min = -10.0, max = 120.0
Selected filters: None

Monoisotopic Mass, Odd and Even Electron Ions
27 formula(e) evaluated with 1 results within limits (up to 51 closest results for each mass)
Elements Used:
C: 0-200 H: 0-400 O: 2-5

eqy36
16:13:27 11-Jun-2012
Voltage EI+

KIB
M120611EA-05AFAMM 7 (0.642)

Autospec Premier
P776
2.78

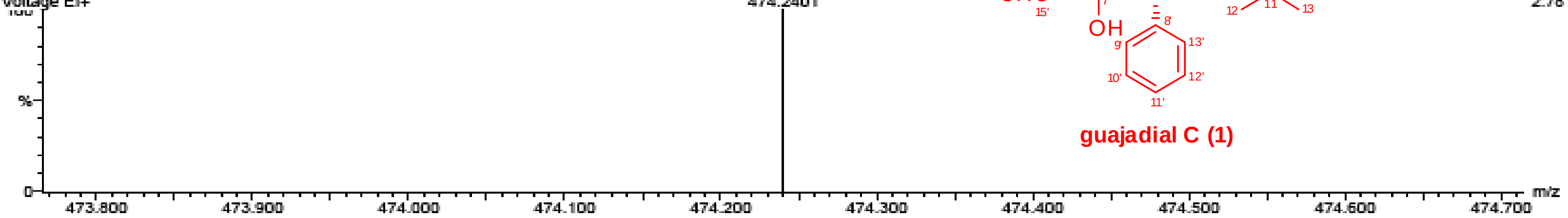


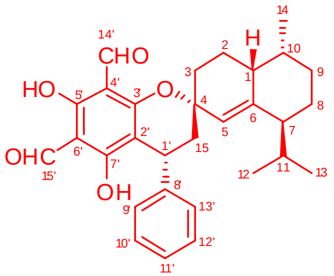


guajadial C (1)

| | | | | | | |
|---|---|---|---|---|---|---|
| Minimum: | | | | -10.0 | | |
| Maximum: | | 100.0 | 10.0 | 120.0 | | |
| Mass | Calc. Mass | mDa | PPM | DBE | i-FIT | Formula |
| 474.2401 | 474.2406 | -0.5 | -1.1 | 14.0 | 5546025.5 | C30 H34 O5 |

S10. IR for guajadial C (**1**)

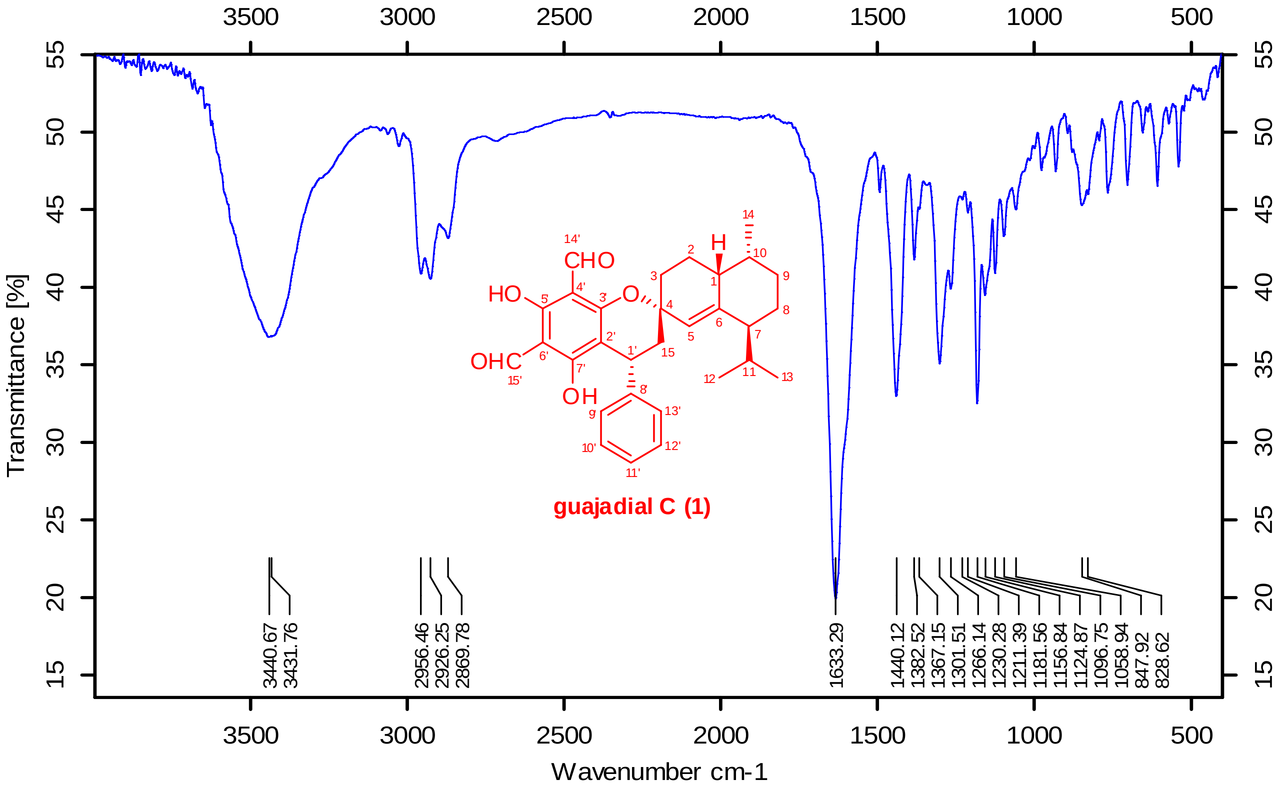


SAMPLE SCANS : 16
DATE : 31/12/2002
TIME : 17:32:08.151 (GMT+1)

SAMPLE : e2787
TECHNIQUE : KBr压片
USER : 120808IR2

S11. $^{1}$H NMR (600MHz) for guajadial D (**2**)

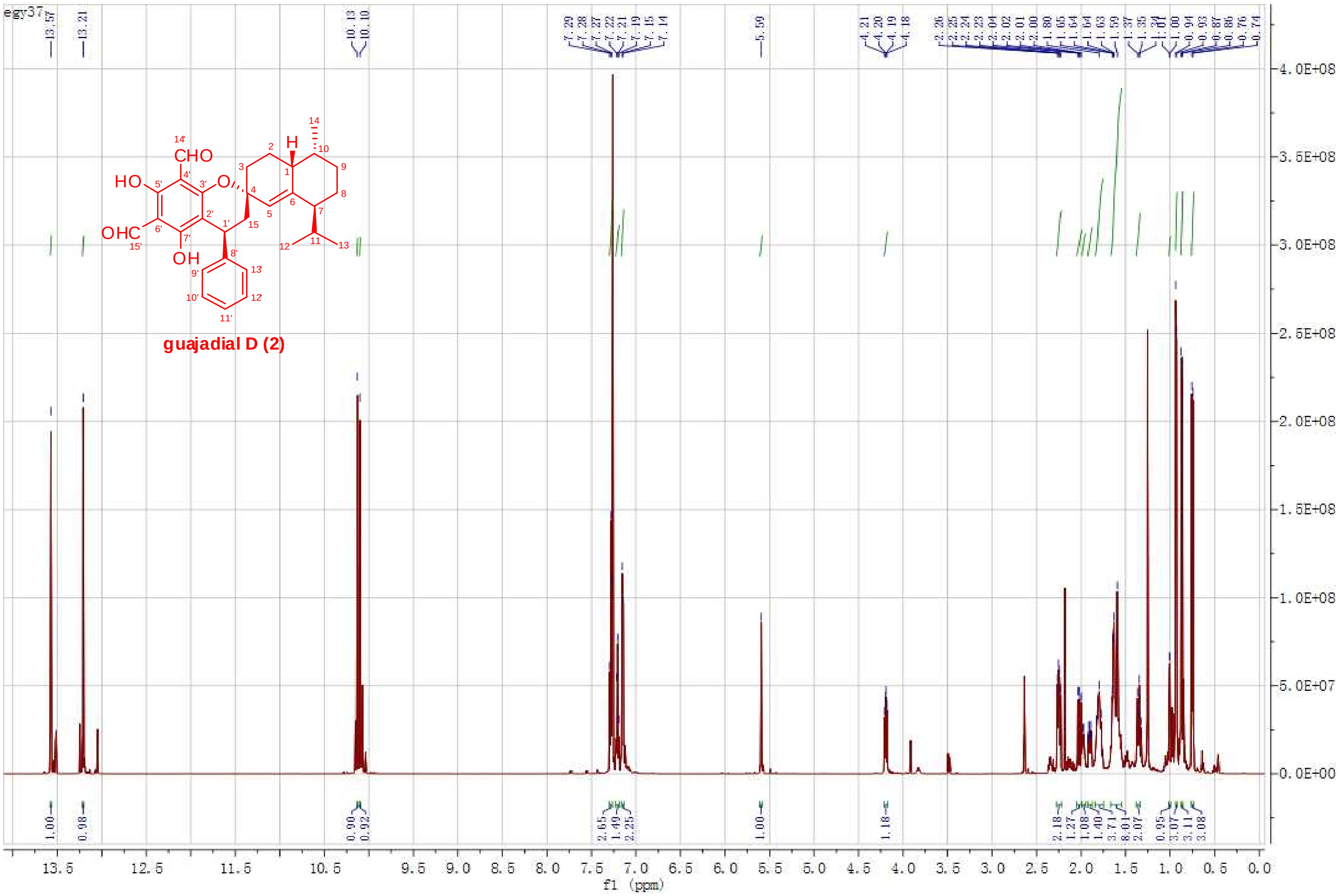

S12. $^{13}$C NMR (DEPT 150 MHz) for guajadial D (**2**)

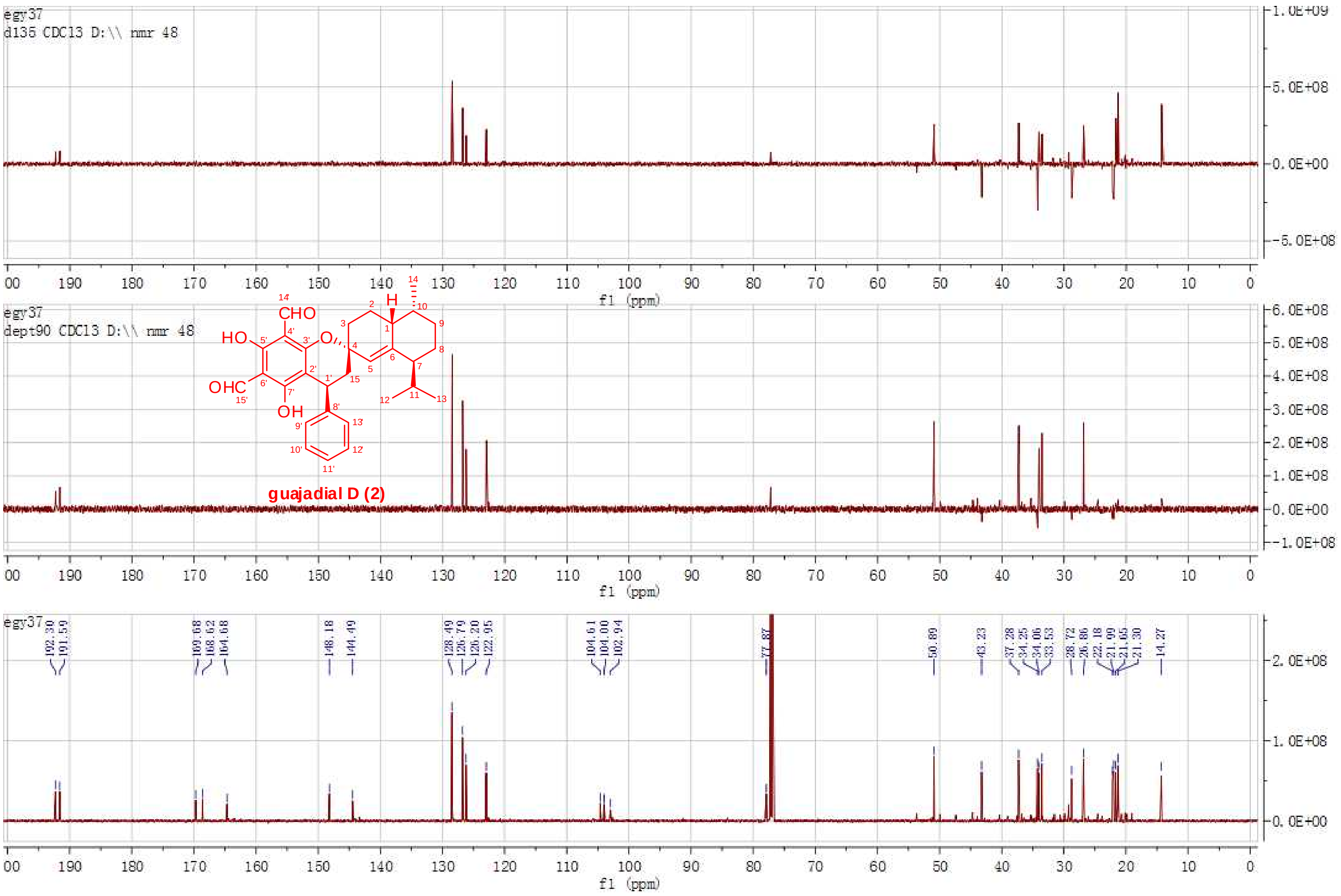

S13. HSQC (600 MHz) for guajadial D (**2**)

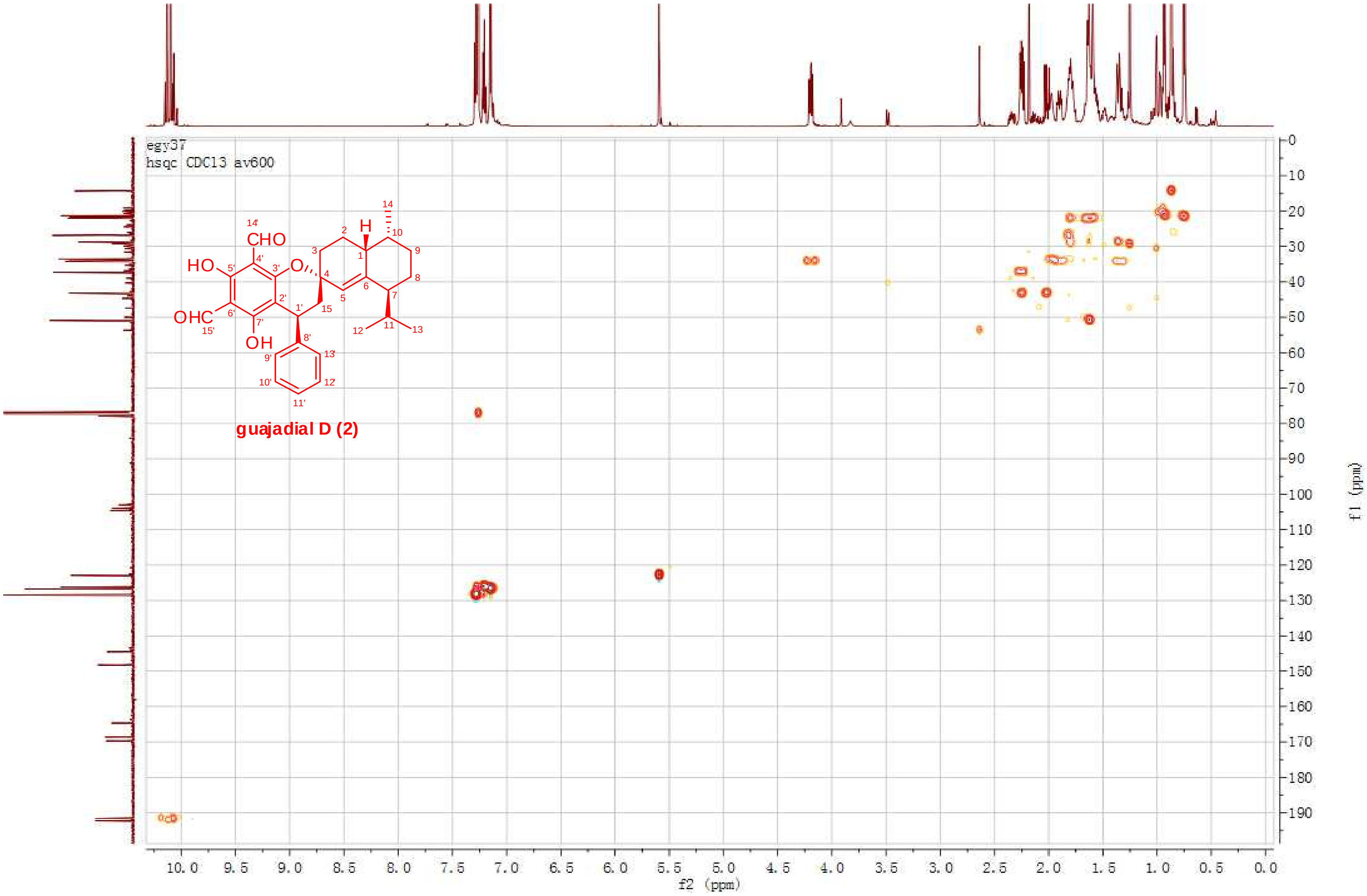

S14. HMBC (600 MHz) for guajadial D (**2**)

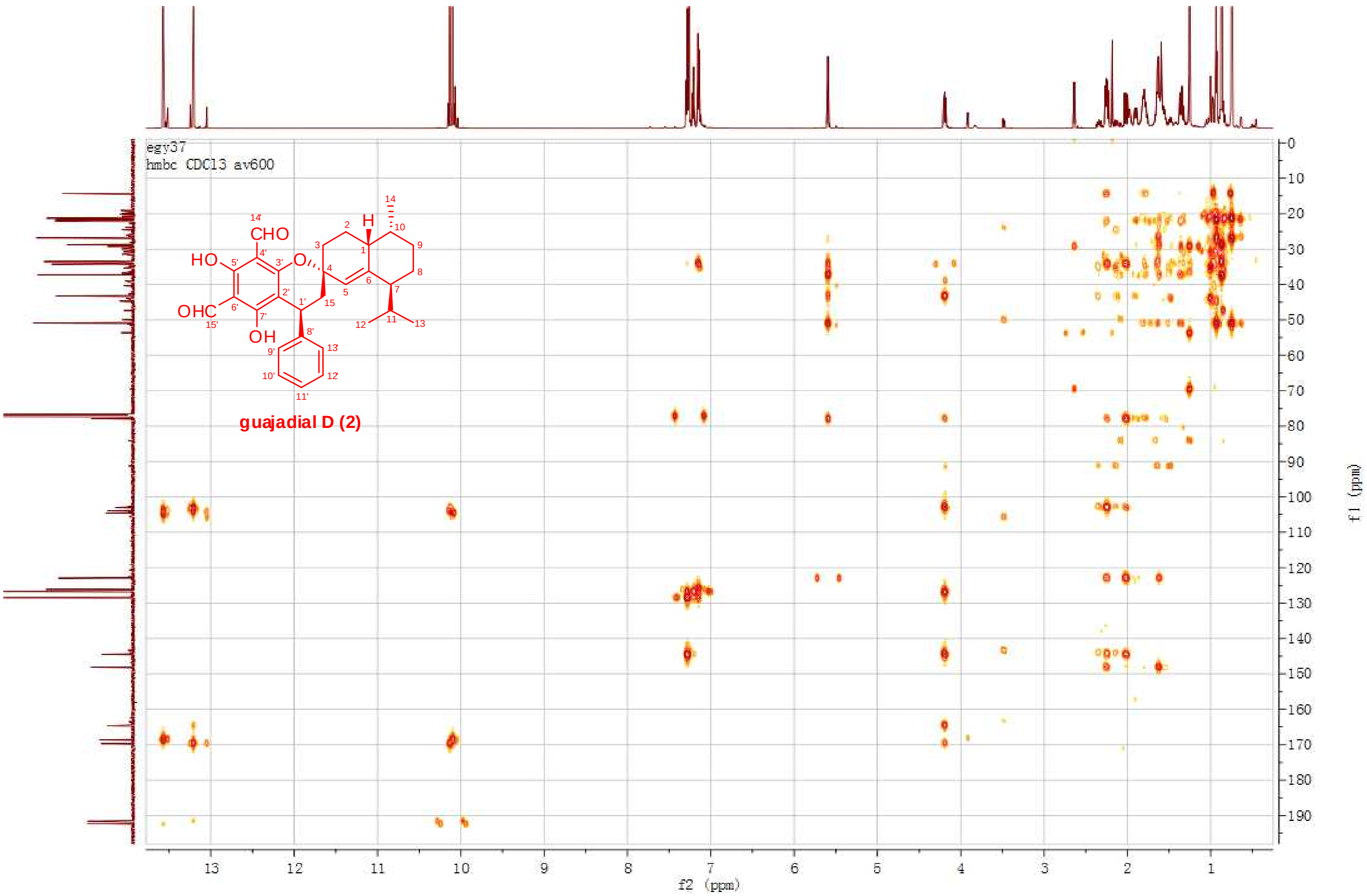

S15. $^{1}H$–$^{1}H$ COSY (600 MHz) for guajadial D (**2**)

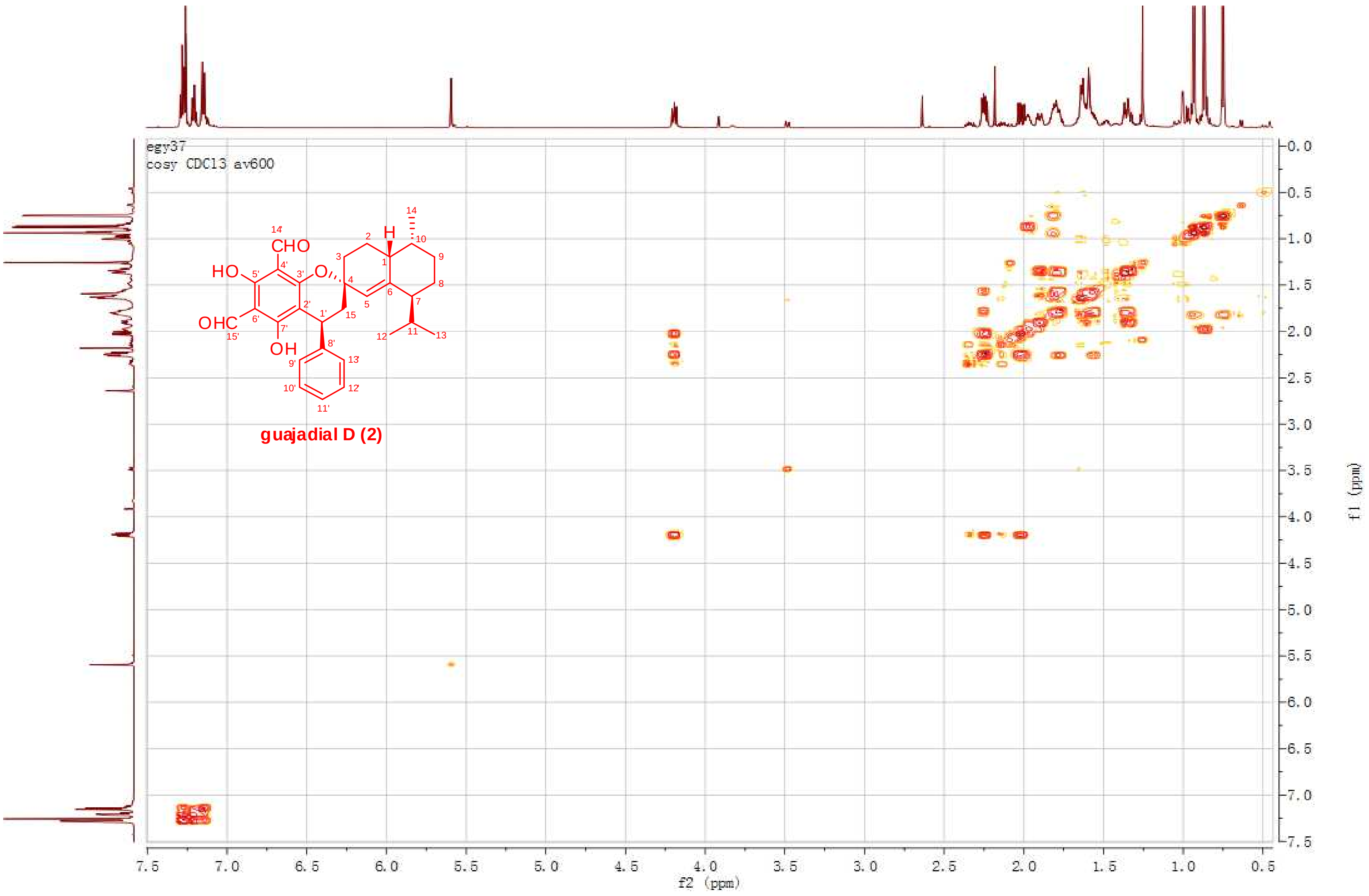

S16. ROESY (600 MHz) for guajadial D (**2**)

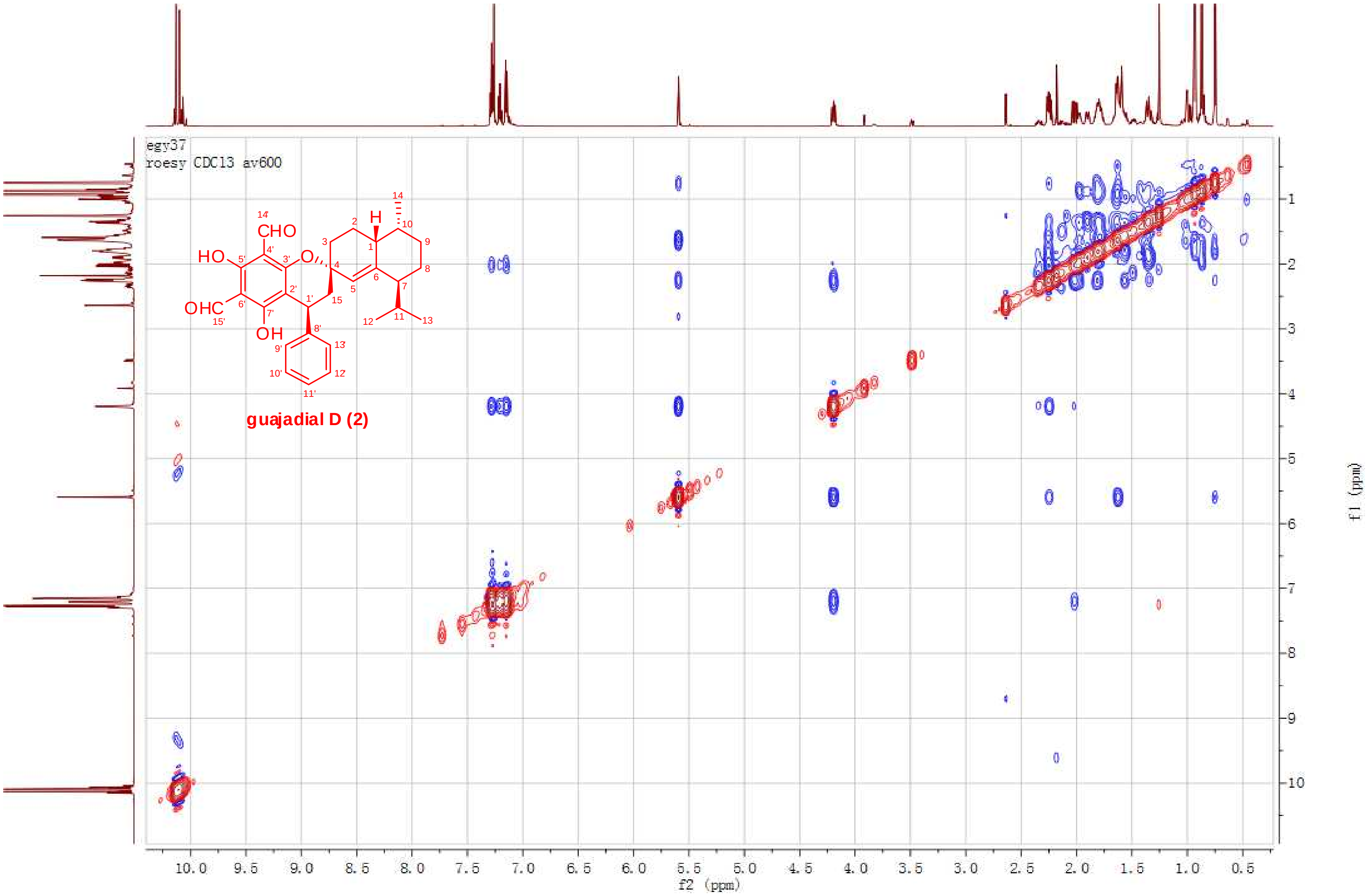

S17. EI-MS for guajadial D (**2**)

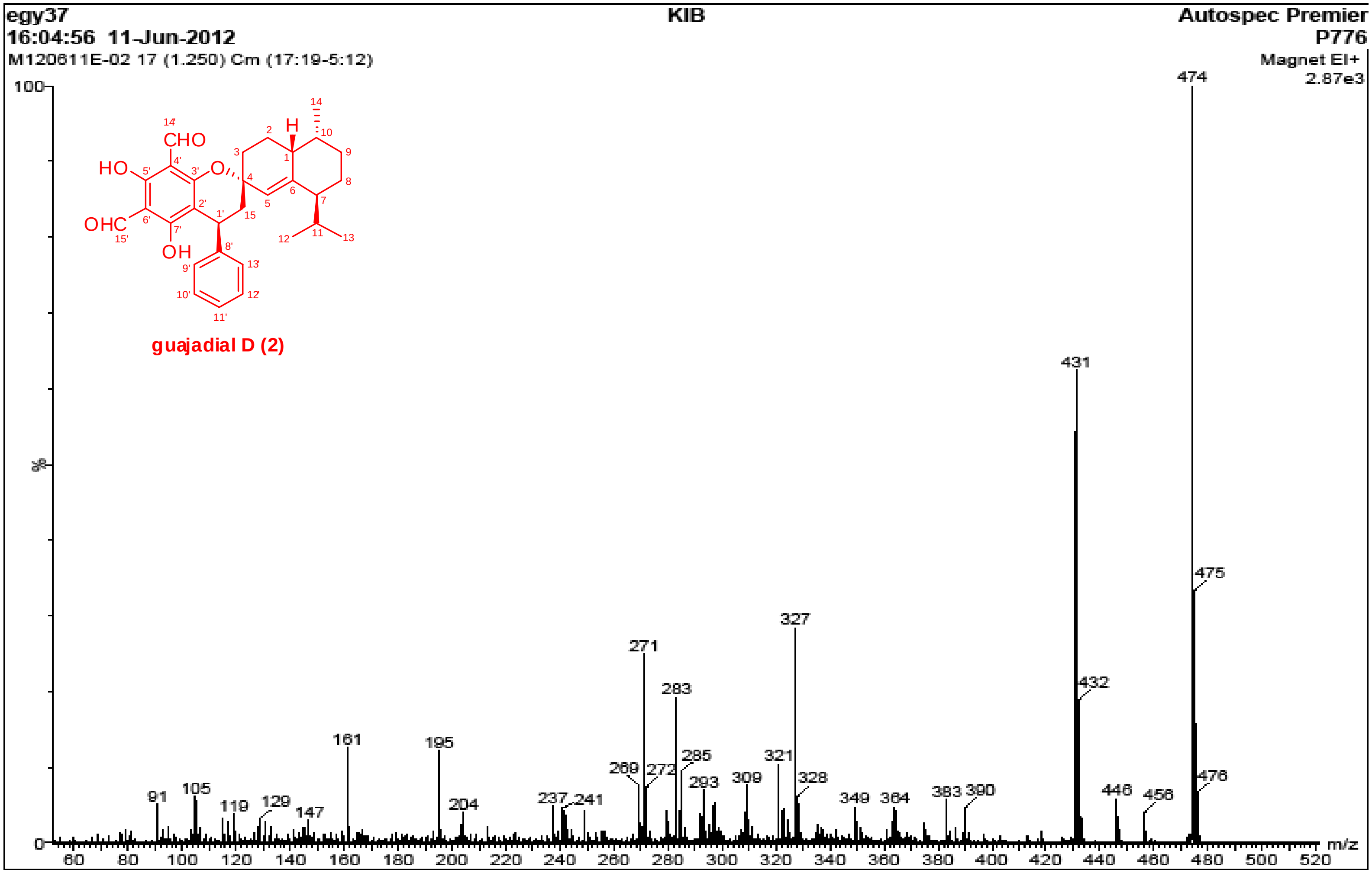

S18. HR-EI-MS for guajadial D (**2**)

**Elemental Composition Report** **Page 1**

**Single Mass Analysis**
Tolerance = 10.0 PPM / DBE: min = -10.0, max = 120.0
Selected filters: None

Monoisotopic Mass, Odd and Even Electron Ions
27 formula(e) evaluated with 1 results within limits (up to 51 closest results for each mass)
Elements Used:
C: 0-200 H: 0-400 O: 2-5

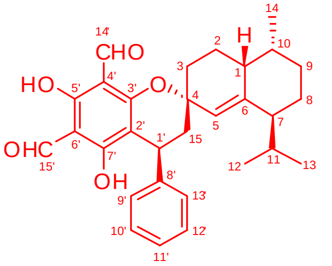


eqy37
16:17:39 11-Jun-2012
Voltage EI+

KIB
M120611EA-04AFAMM 16 (1.469)

Autospec Premier
P776
1.40e3

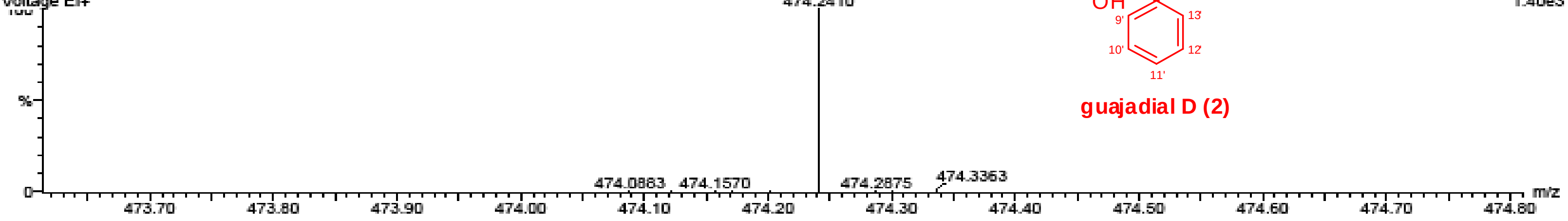


| | | | | | | |
|---|---|---|---|---|---|---|
| Minimum: | | | | -10.0 | | |
| Maximum: | | 100.0 | 10.0 | 120.0 | | |
| Mass | Calc. Mass | mDa | PPM | DBE | i-FIT | Formula |
| 474.2410 | 474.2406 | 0.4 | 0.8 | 14.0 | 5546717.5 | C30 H34 O5 |

S19. IR for guajadial D (**2**)

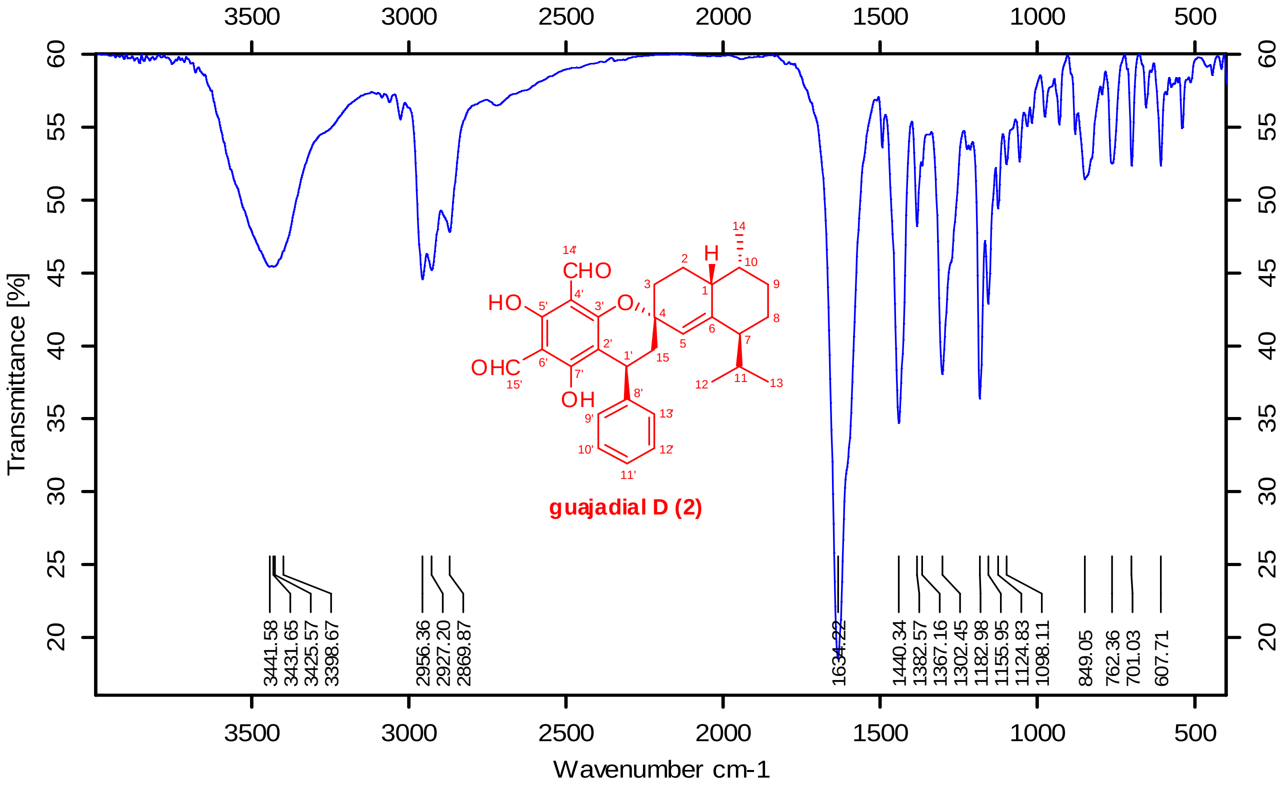


SAMPLE SCANS : 16
DATE : 31/12/2002
TIME : 17:23:07.501 (GMT+1)

SAMPLE : e2788
TECHNIQUE : KBr压片
USER : 120808IR1

S20. $^{1}$H NMR (600MHz) for guajadial E (**3**)

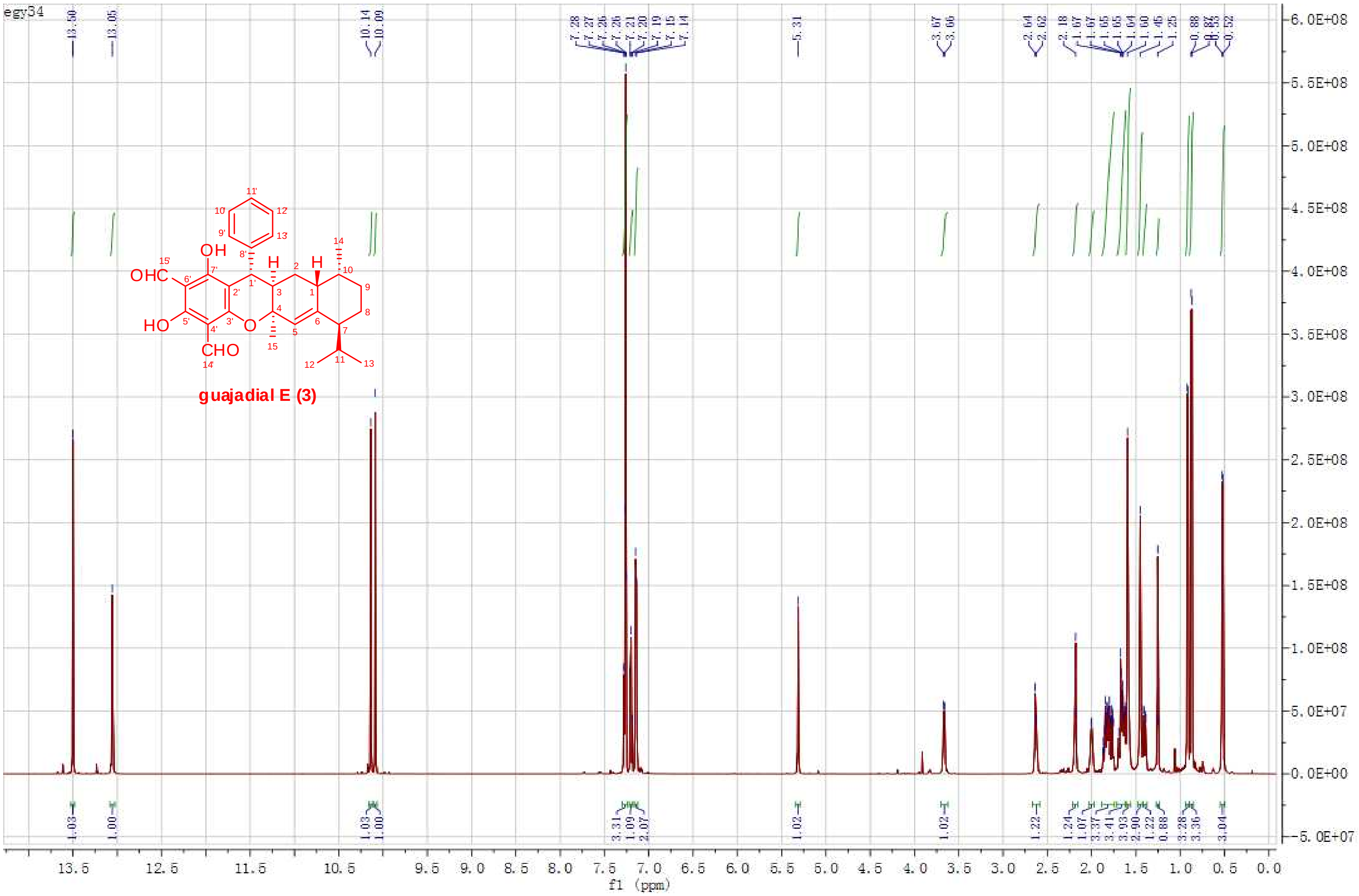

S21. $^{13}$C NMR (DEPT 150 MHz) for guajadial E (**3**)

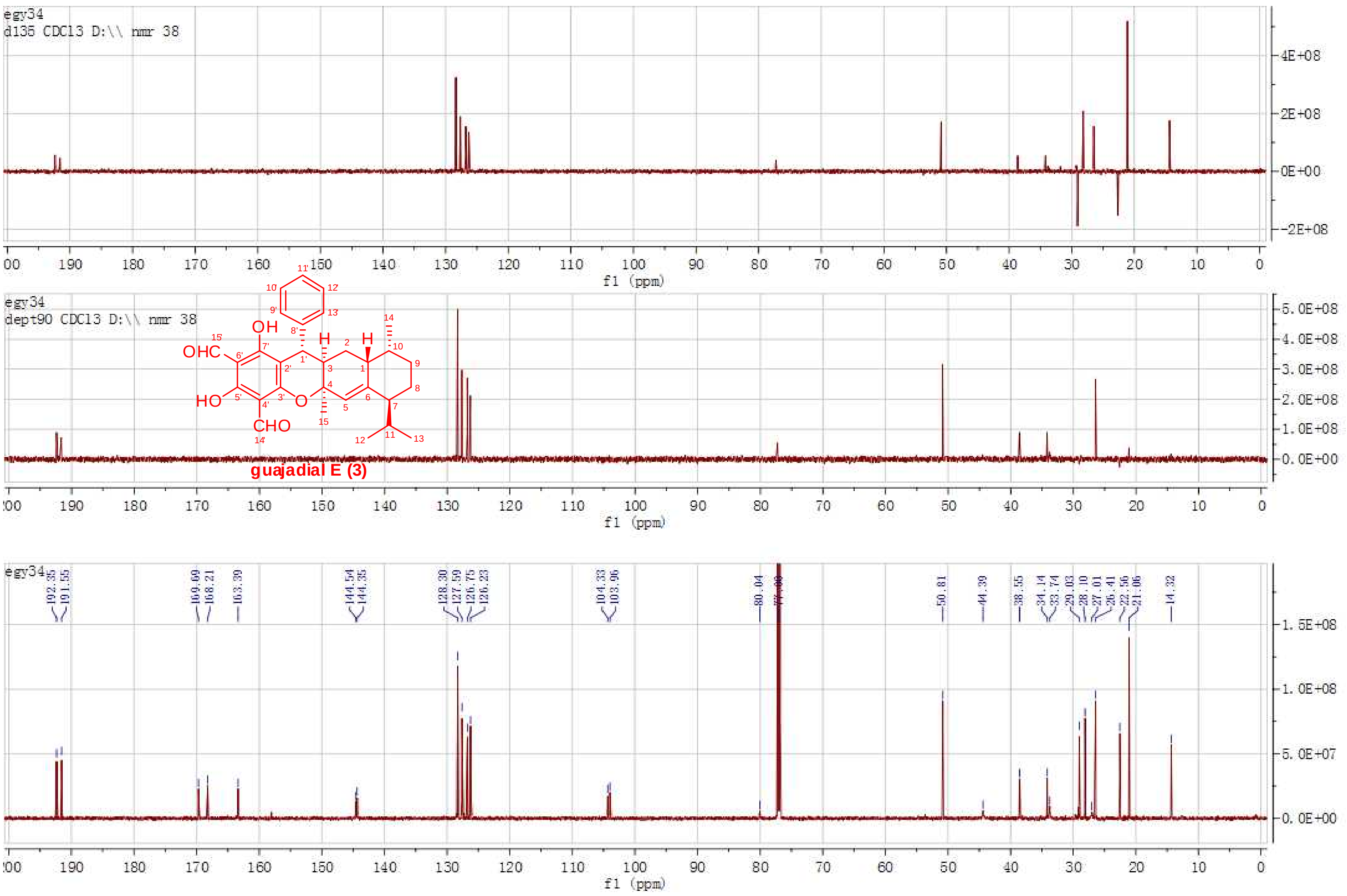

S22. HSQC (600 MHz) for guajadial E (**3**)

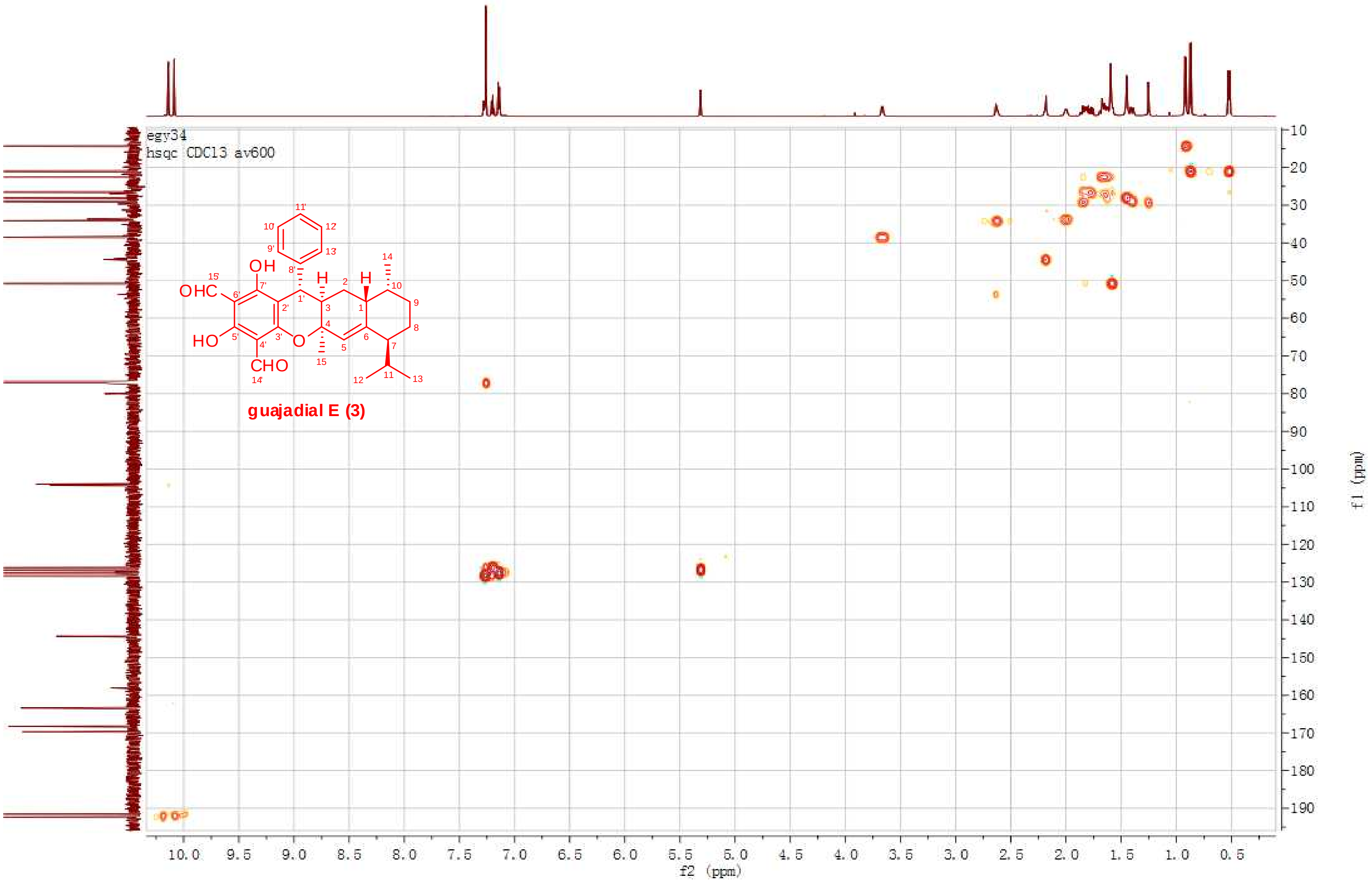

S23. HMBC (600 MHz) for guajadial E (**3**)

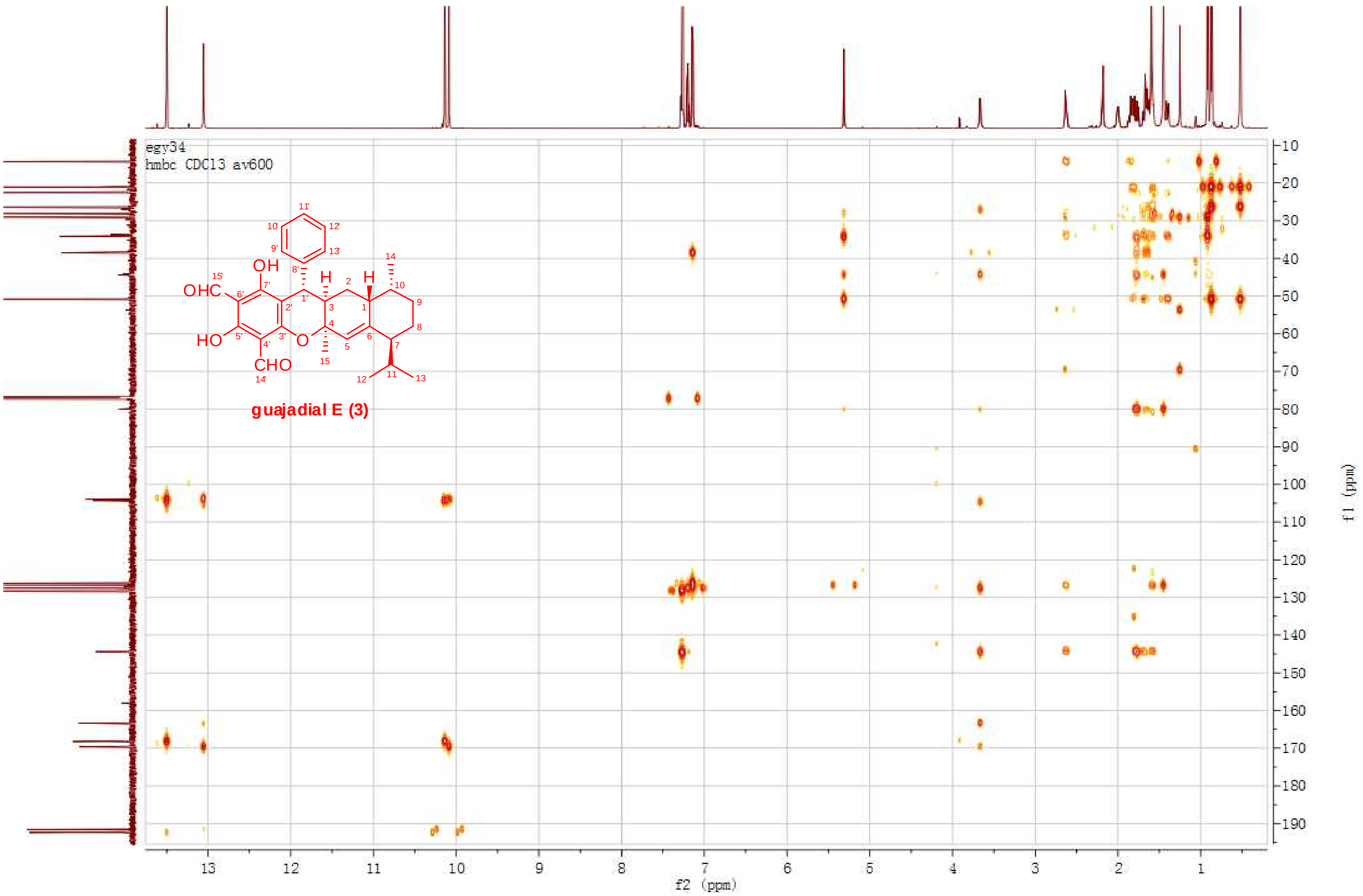

S24. $^1$H–$^1$H COSY (600 MHz) for guajadial E (**3**)

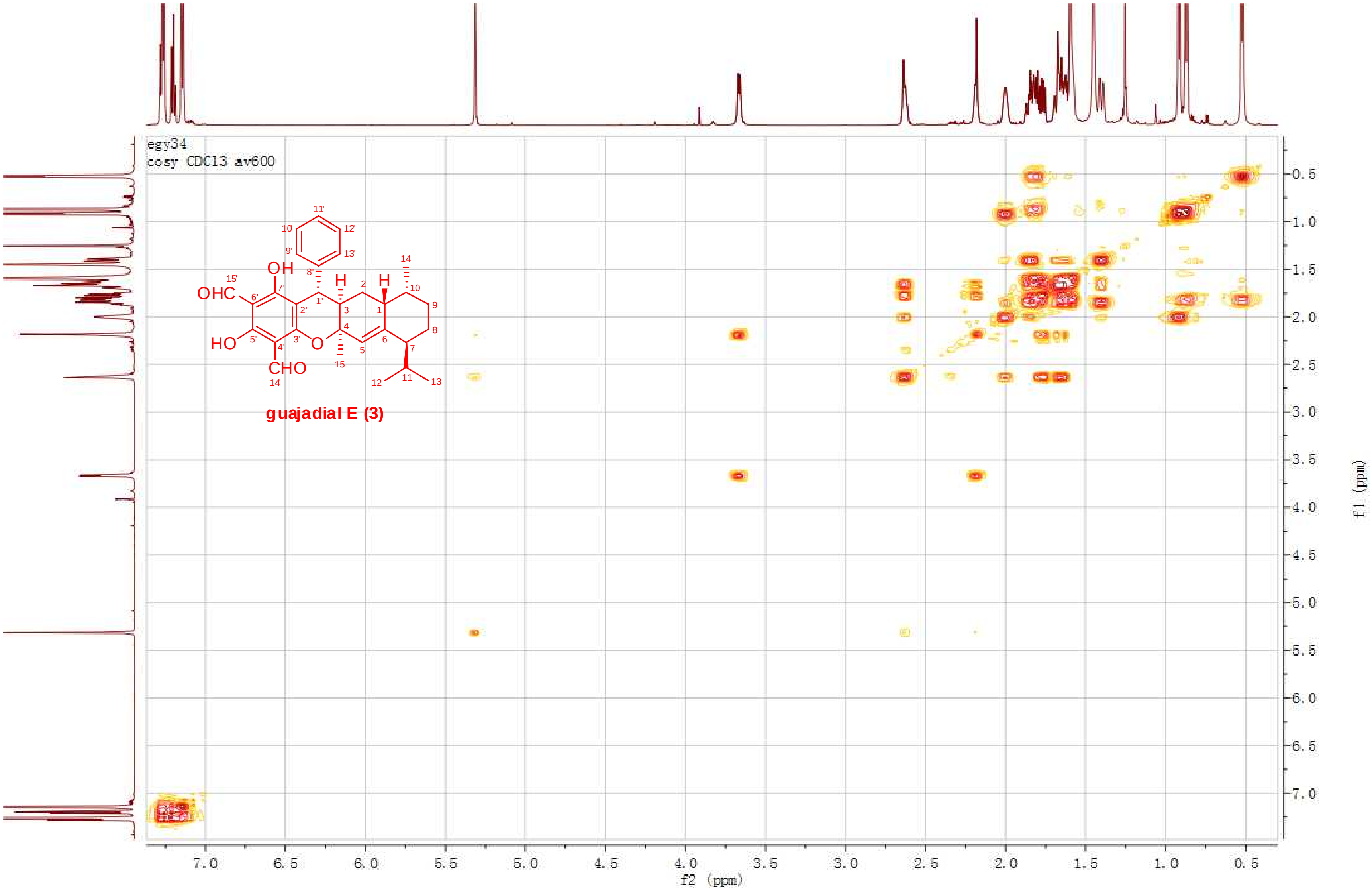

S25. ROESY (600 MHz) for guajadial E (**3**)

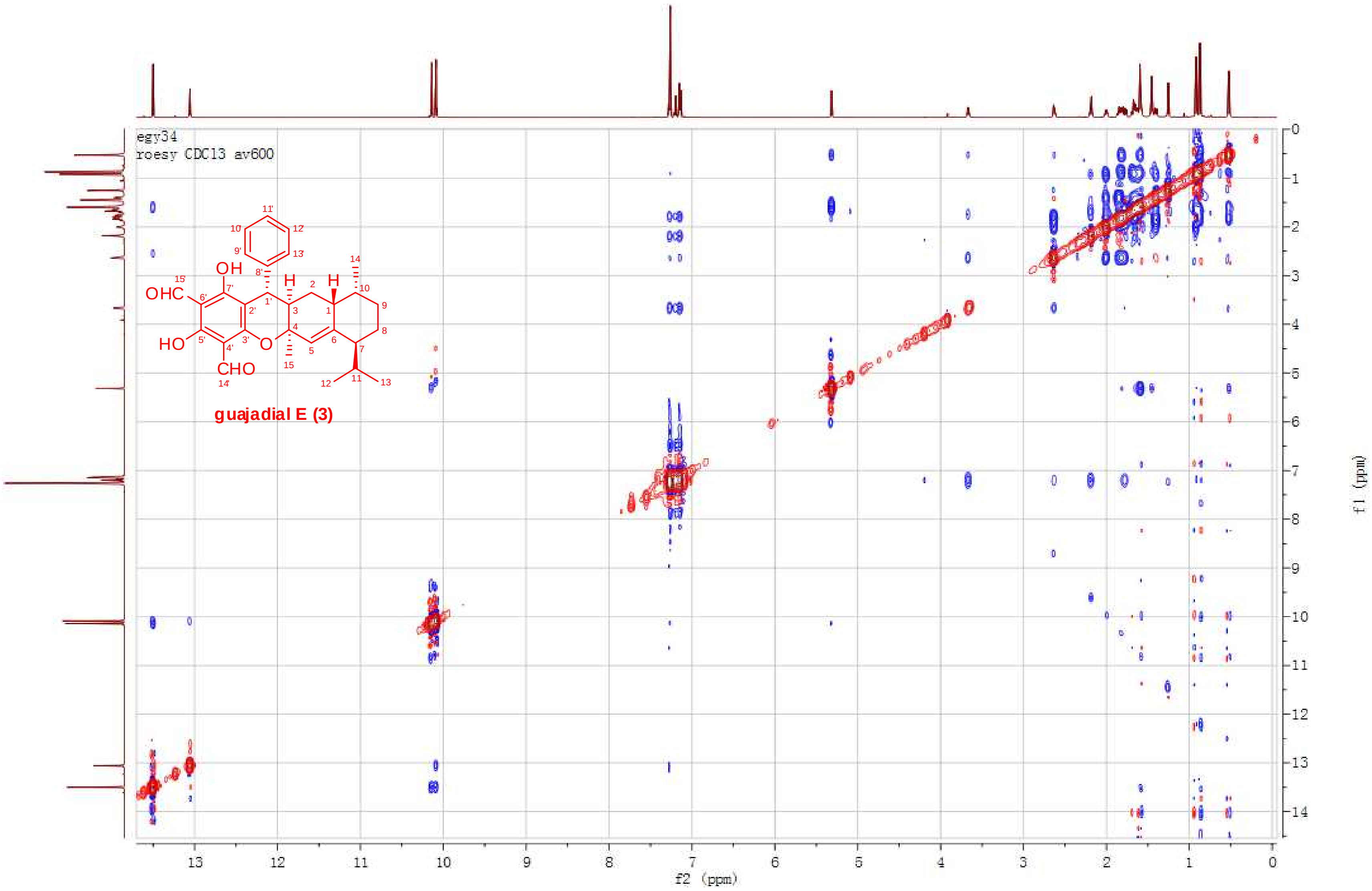

S26. EI-MS for guajadial E (**3**)

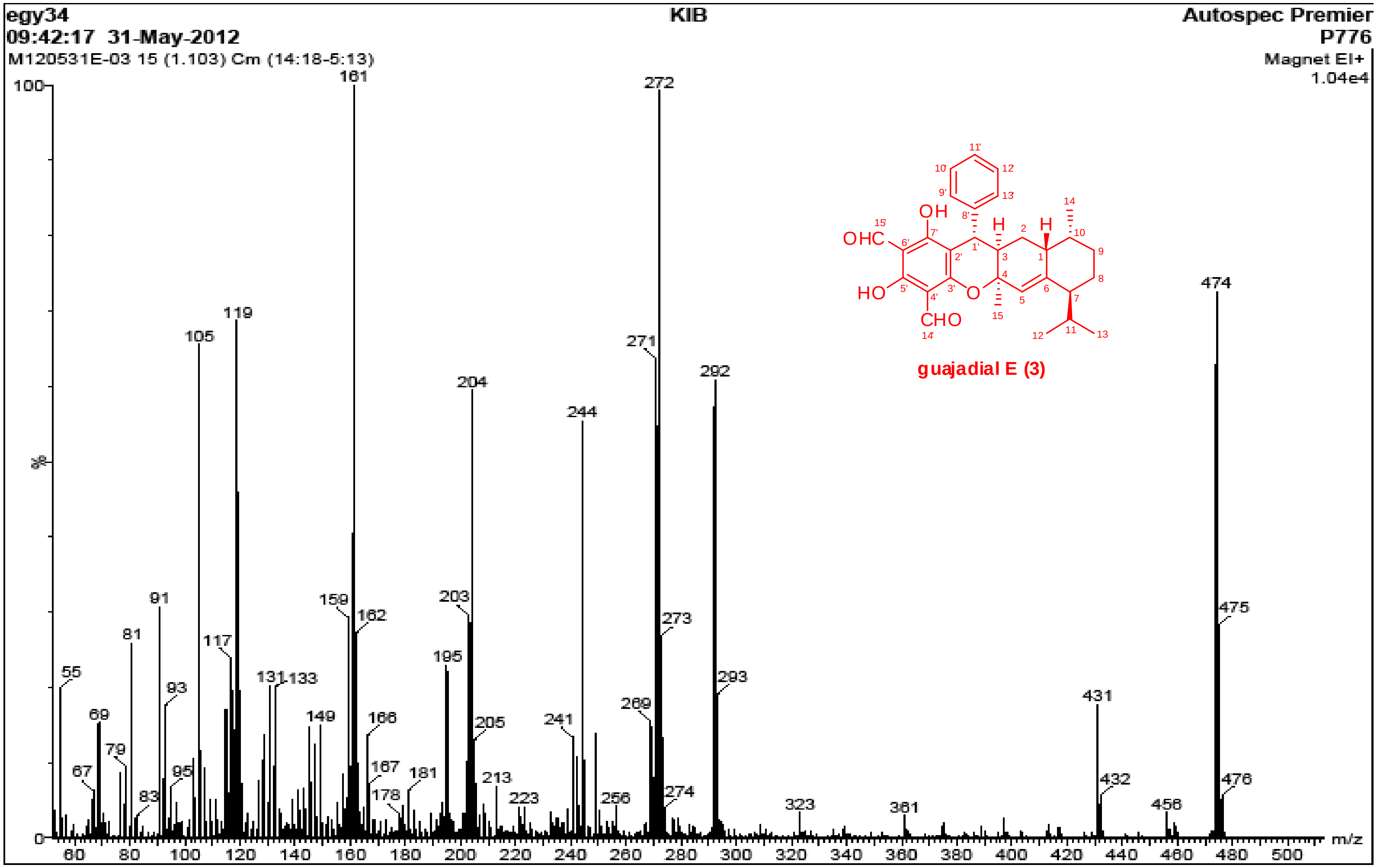

S27. HR-EI-MS for guajadial E (**3**)

**Elemental Composition Report** **Page 1**

**Single Mass Analysis**
Tolerance = 10.0 PPM / DBE: min = -10.0, max = 120.0
Selected filters: None

Monoisotopic Mass, Odd and Even Electron Ions
27 formula(e) evaluated with 1 results within limits (up to 51 closest results for each mass)
Elements Used:
C: 0-200 H: 0-400 O: 2-5

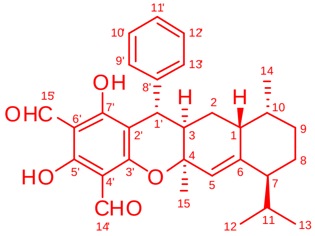


guajadial E (3)

eqy34
09:45:29 31-May-2012
Voltage EI+

KIB
M120531EA-02AFAMM 20 (1.836)

Autospec Premier
P776
2.27e3

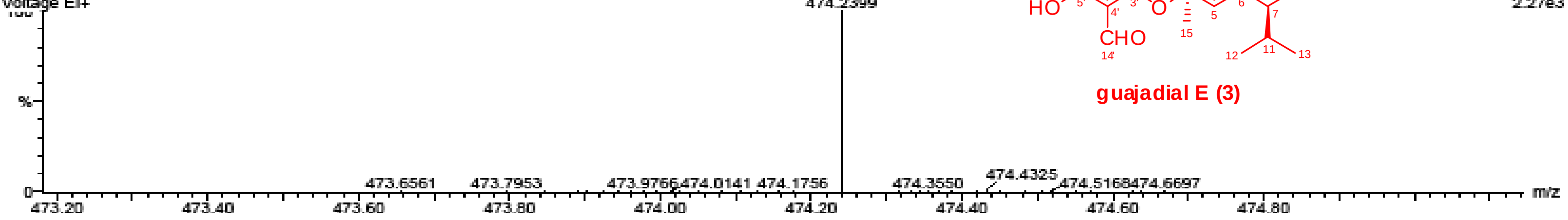


| Mass | Calc. Mass | mDa | PPM | DBE | i-FIT | Formula |
|---|---|---|---|---|---|---|
| Minimum: | | | | -10.0 | | |
| Maximum: | | 100.0 | 10.0 | 120.0 | | |
| 474.2399 | 474.2406 | -0.7 | -1.5 | 14.0 | 5547152.0 | C30 H34 O5 |

S28. IR for guajadial E (**3**)

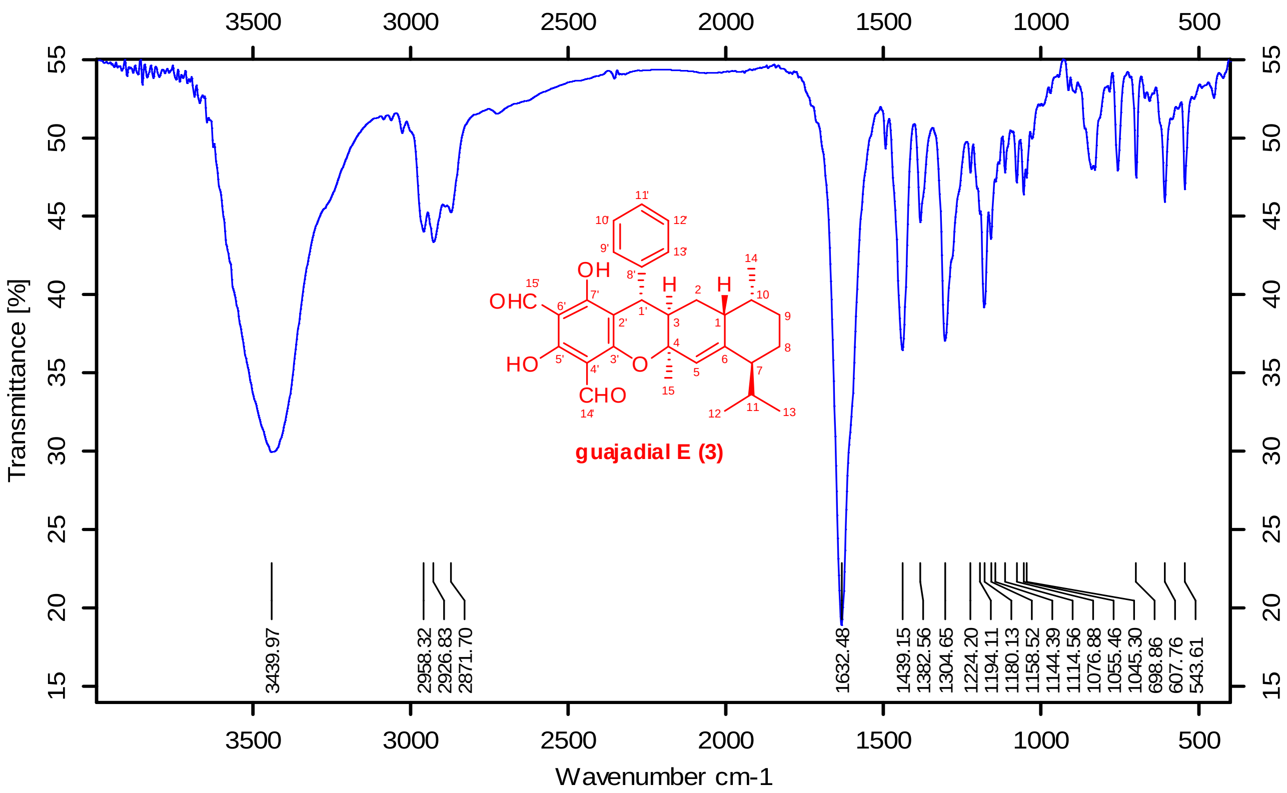


SAMPLE SCANS : 16
DATE : 31/12/2002
TIME : 17:47:03.401 (GMT+1)

SAMPLE : e2776
TECHNIQUE : KBr压片
USER : 120808IR4

S29. $^1$H NMR (600MHz) for guajadial F (**4**)

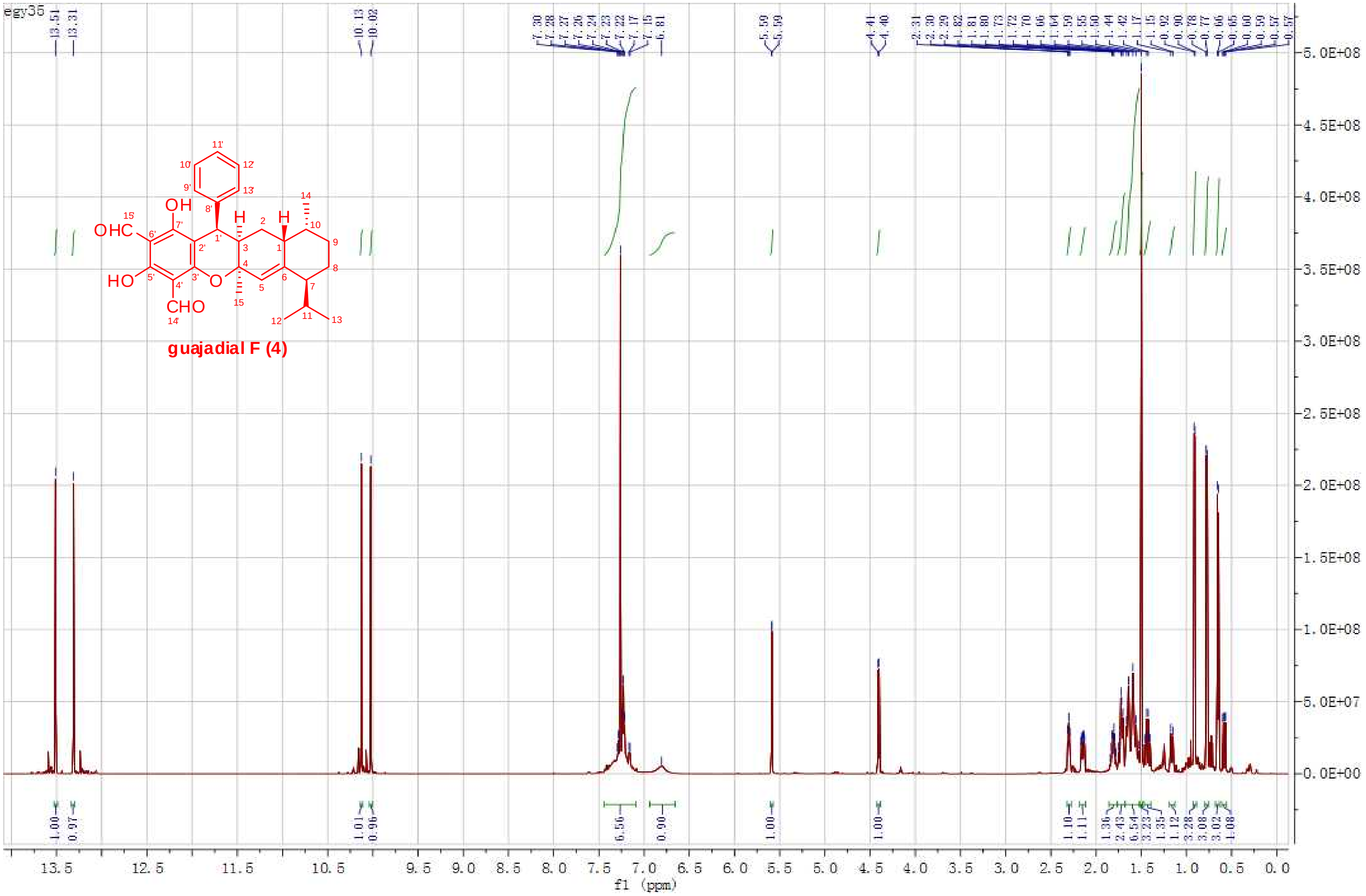

S30. $^{13}$C NMR (DEPT 150 MHz) for guajadial F (**4**)

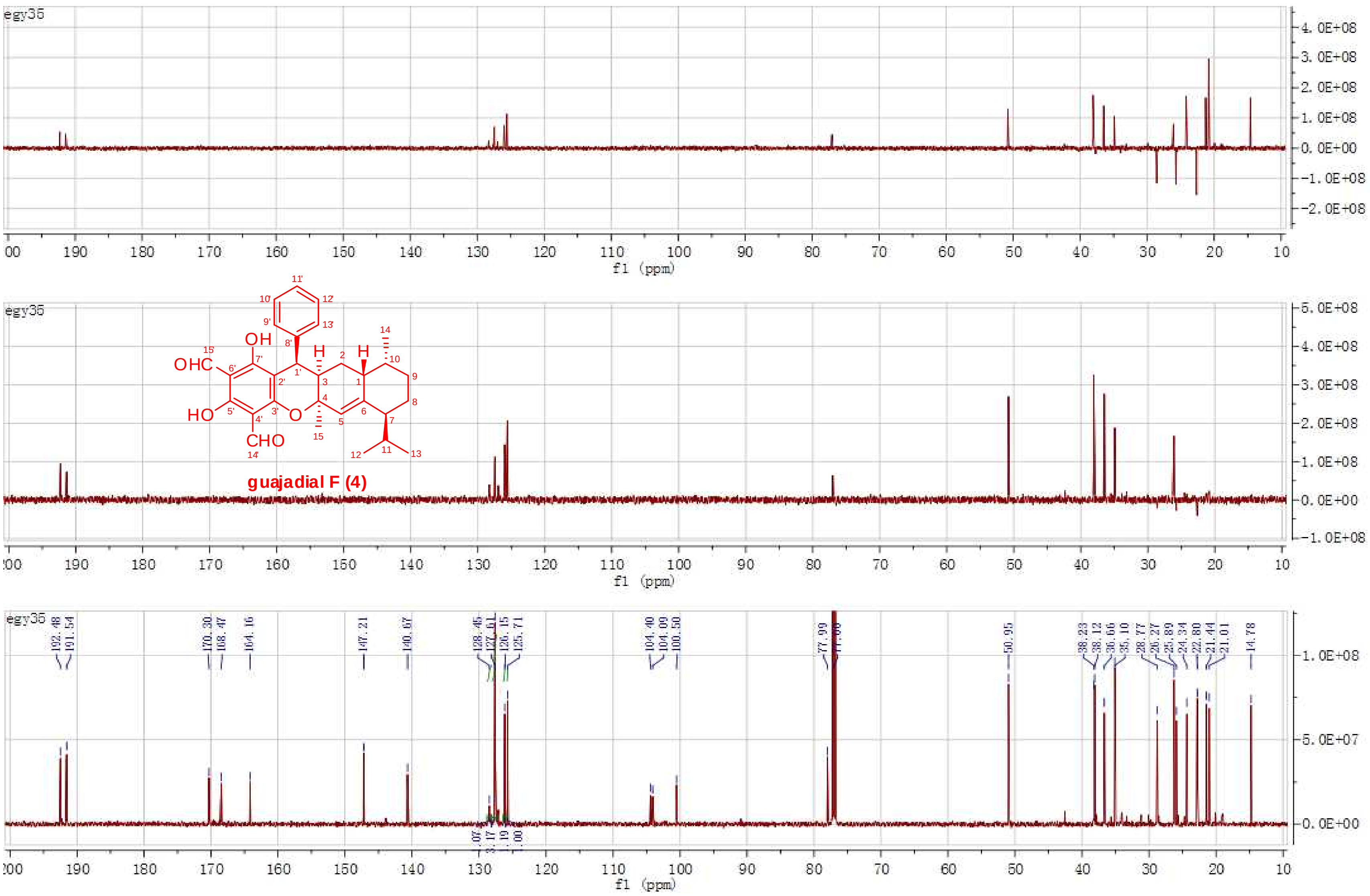

S31. The relative areas of peaks of overlapping carbon signals for guajadial F (**4**)

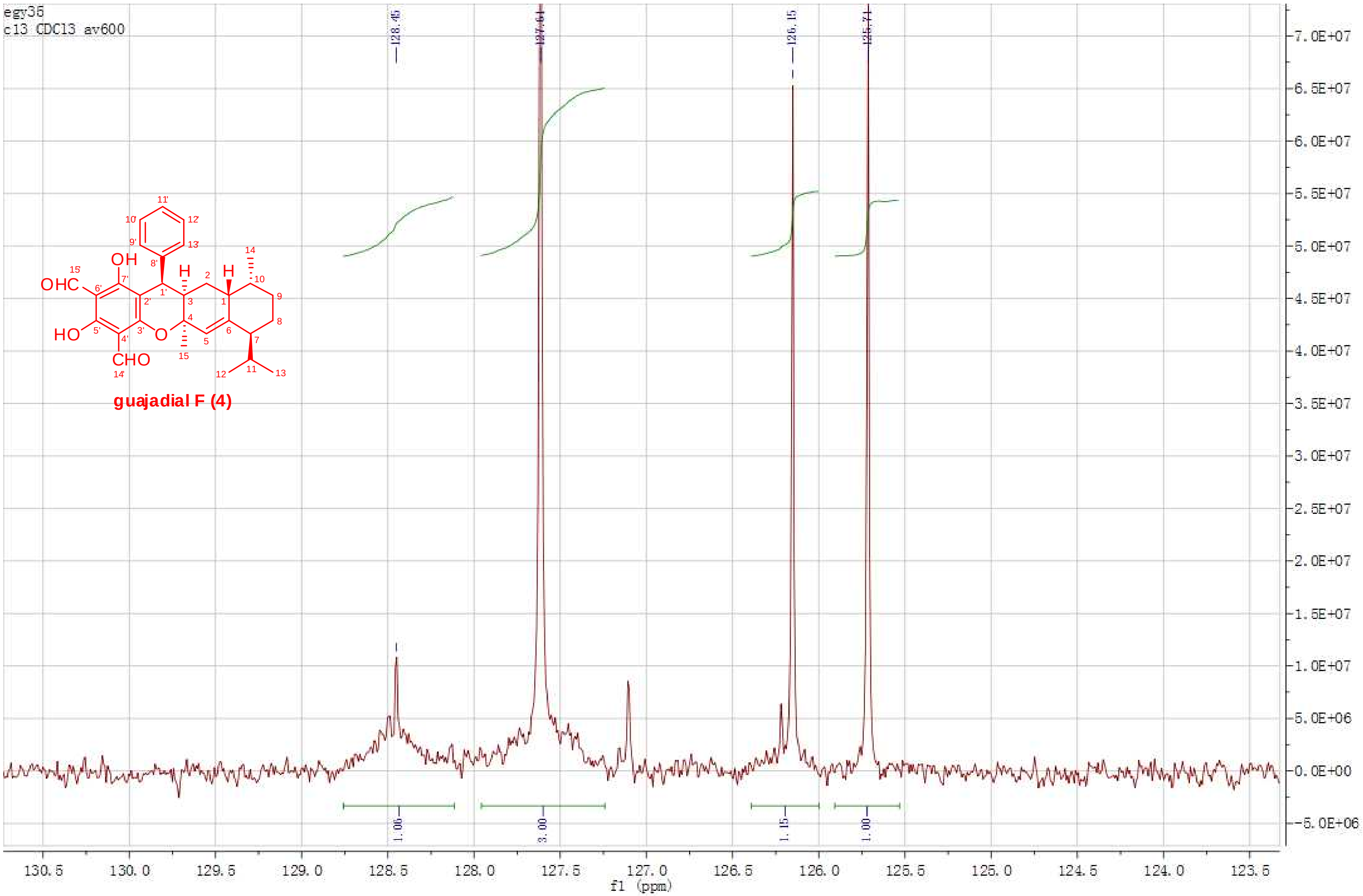

S32. HSQC (600 MHz) for guajadial F (**4**)

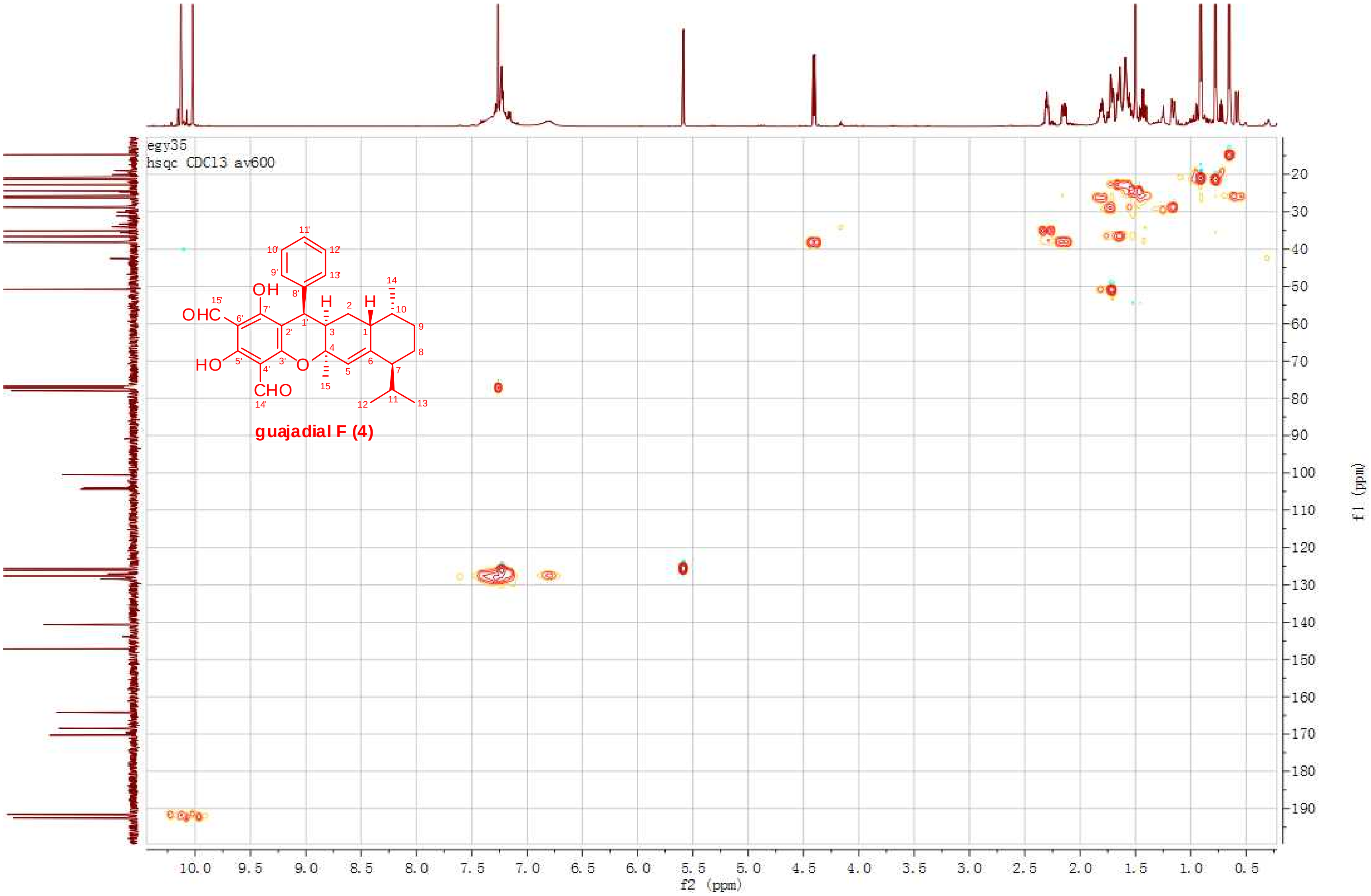

S33. HMBC (600 MHz) for guajadial F (**4**)

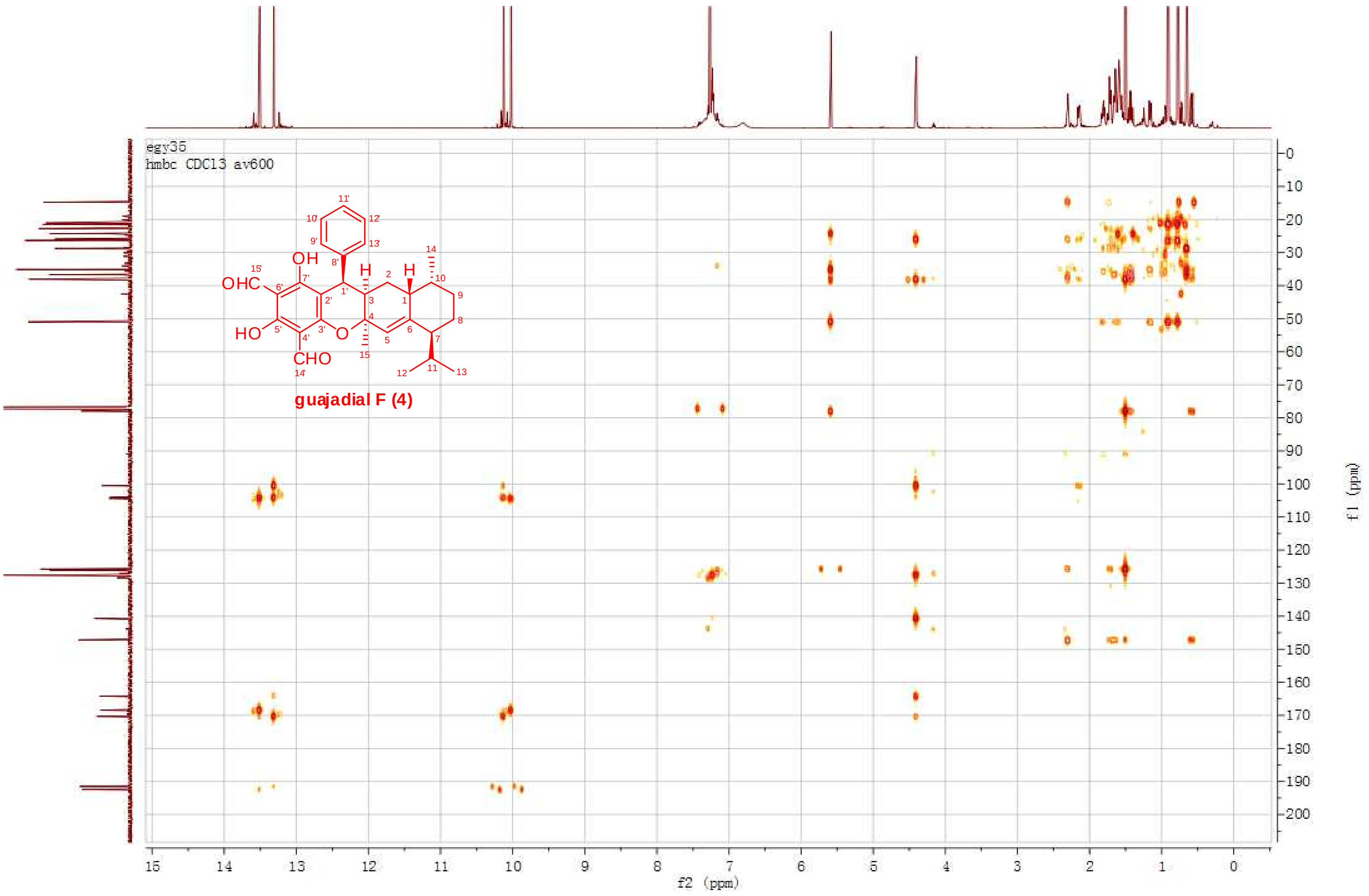

S34. $^1$H–$^1$H COSY (600 MHz) for guajadial F (**4**)

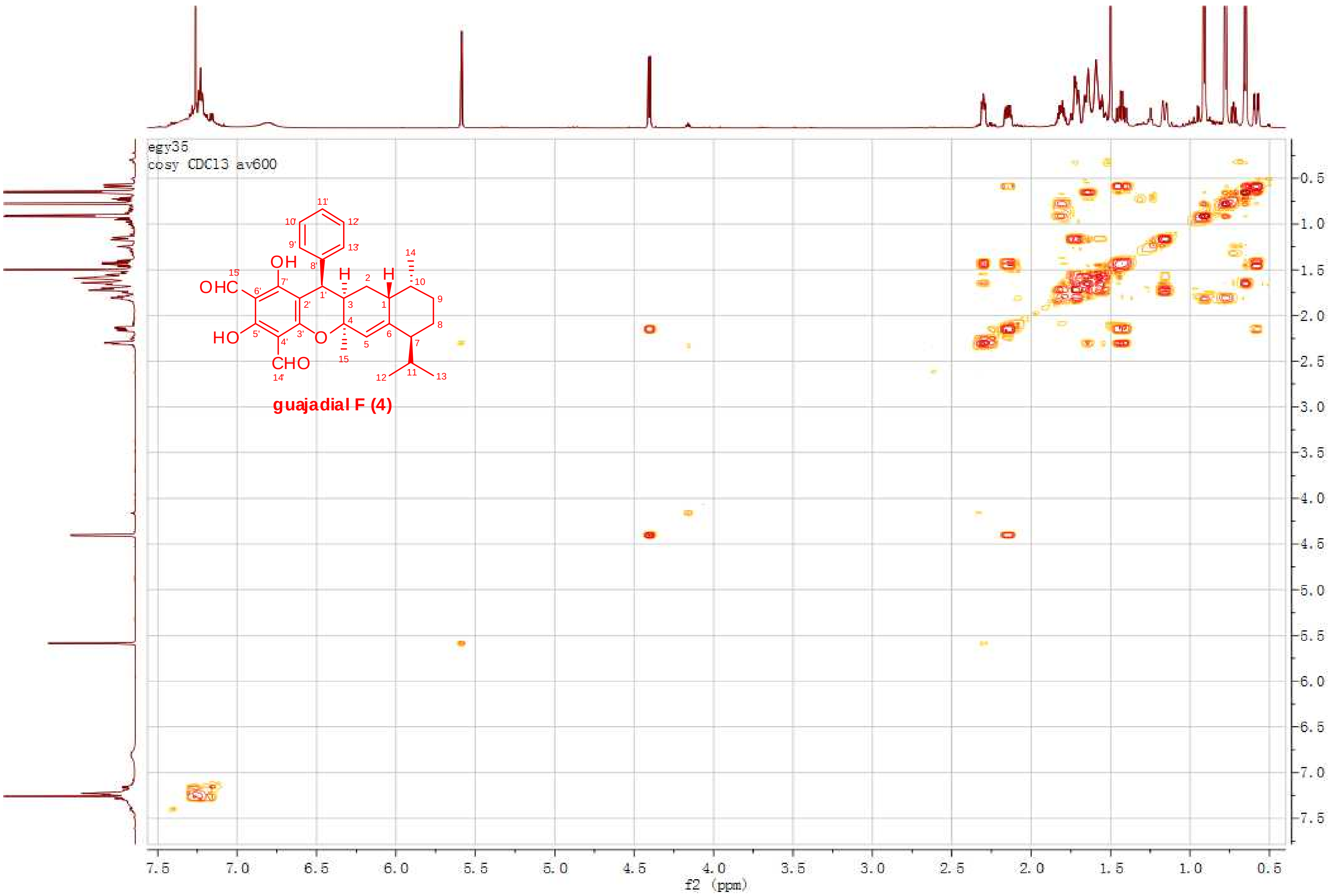

S35. ROESY (600 MHz) for guajadial F (**4**)

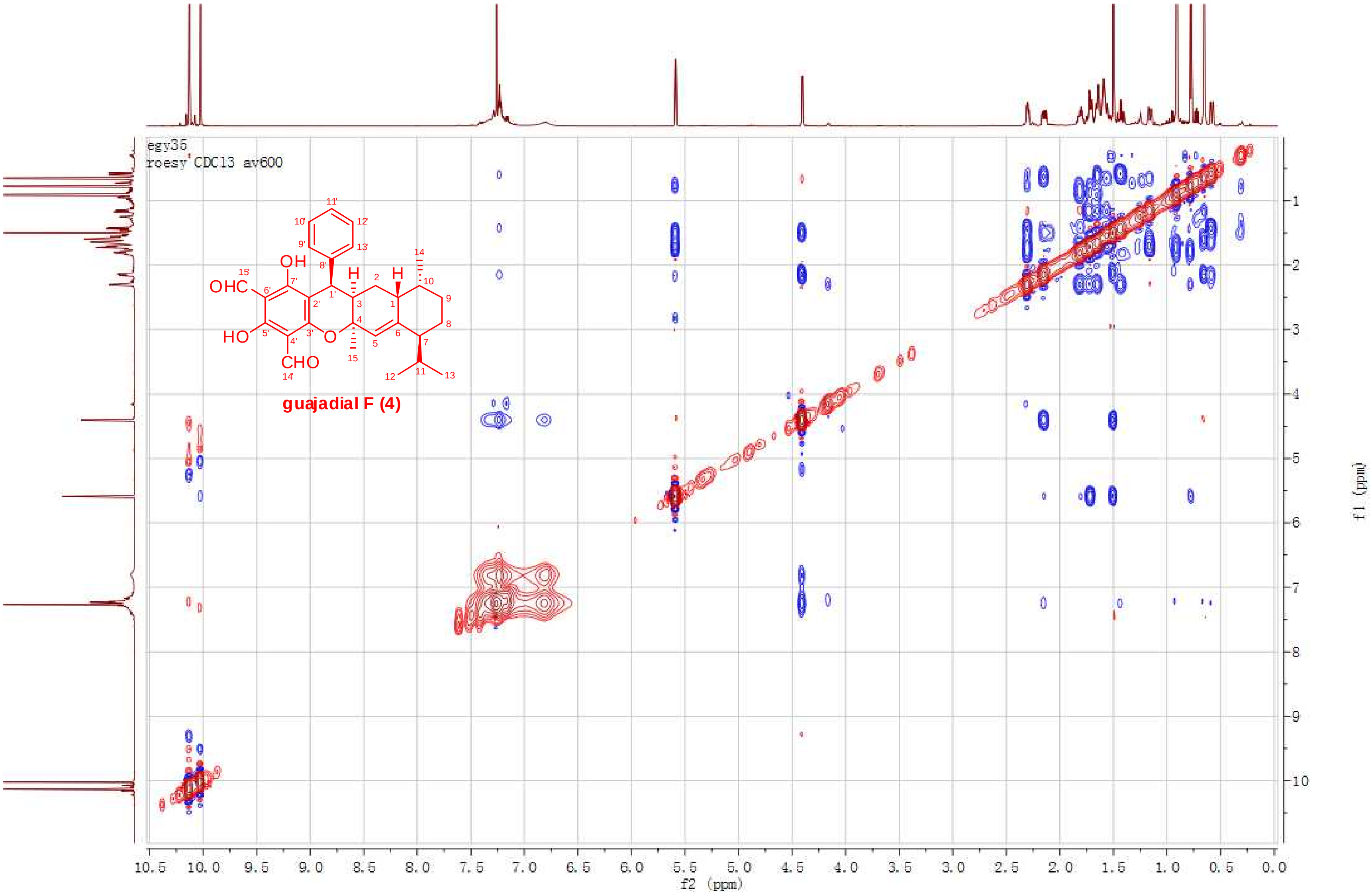

S36. EI-MS for guajadial F (**4**)

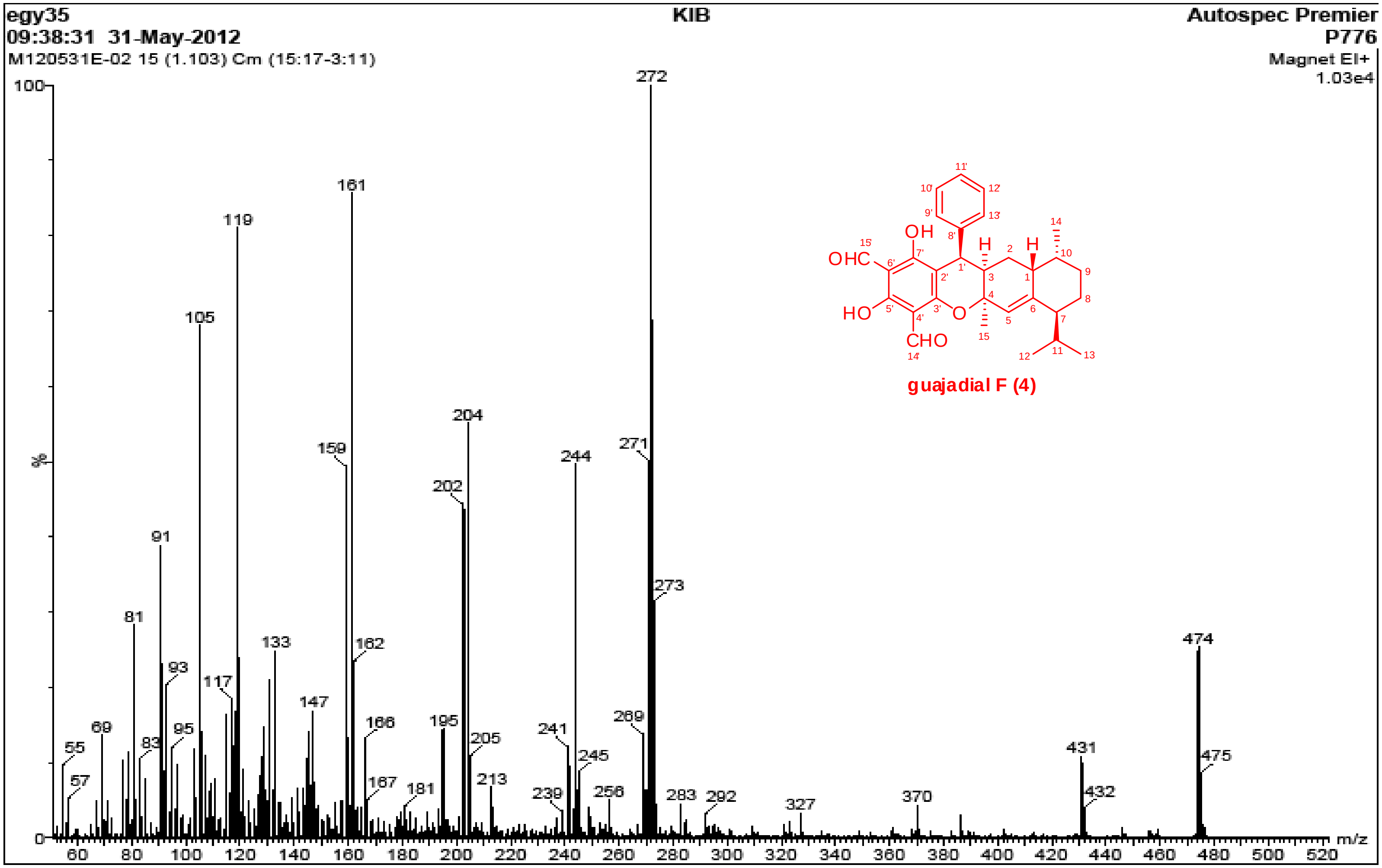

S37. HR-EI-MS for guajadial F (**4**)

## Elemental Composition Report

Page 1

### Single Mass Analysis

Tolerance = 10.0 PPM / DBE: min = -10.0, max = 120.0
Selected filters: None

Monoisotopic Mass, Odd and Even Electron Ions
27 formula(e) evaluated with 1 results within limits (up to 51 closest results for each mass)
Elements Used:
C: 0-200 H: 0-400 O: 2-5

eqy35
09:51:55 31-May-2012
Voltage EI+

KIB
M120531EA-01AFAMM 22 (2.020)

Autospec Premier
P776
181

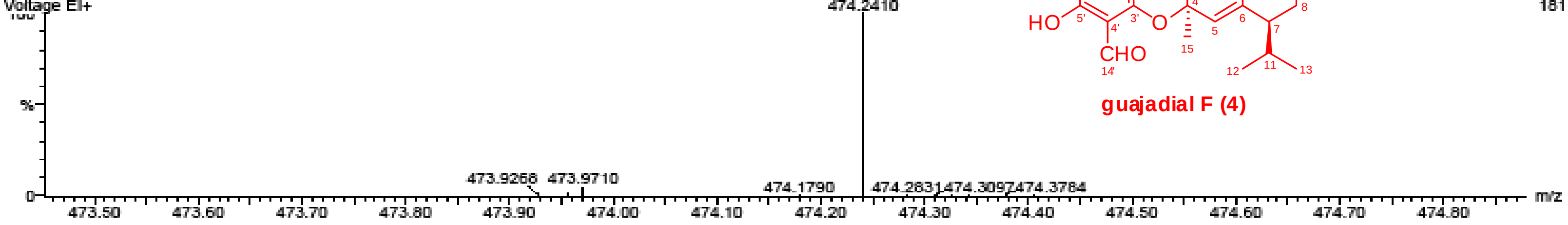


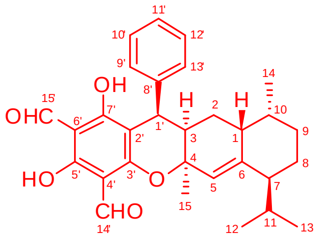


guajadial F (4)

| | | | | | | |
|---|---|---|---|---|---|---|
| Minimum: | | | | -10.0 | | |
| Maximum: | | 100.0 | 10.0 | 120.0 | | |
| Mass | Calc. Mass | mDa | PPM | DBE | i-FIT | Formula |
| 474.2410 | 474.2406 | 0.4 | 0.8 | 14.0 | 5546108.5 | C30 H34 O5 |

S38. IR for guajadial F (**4**)

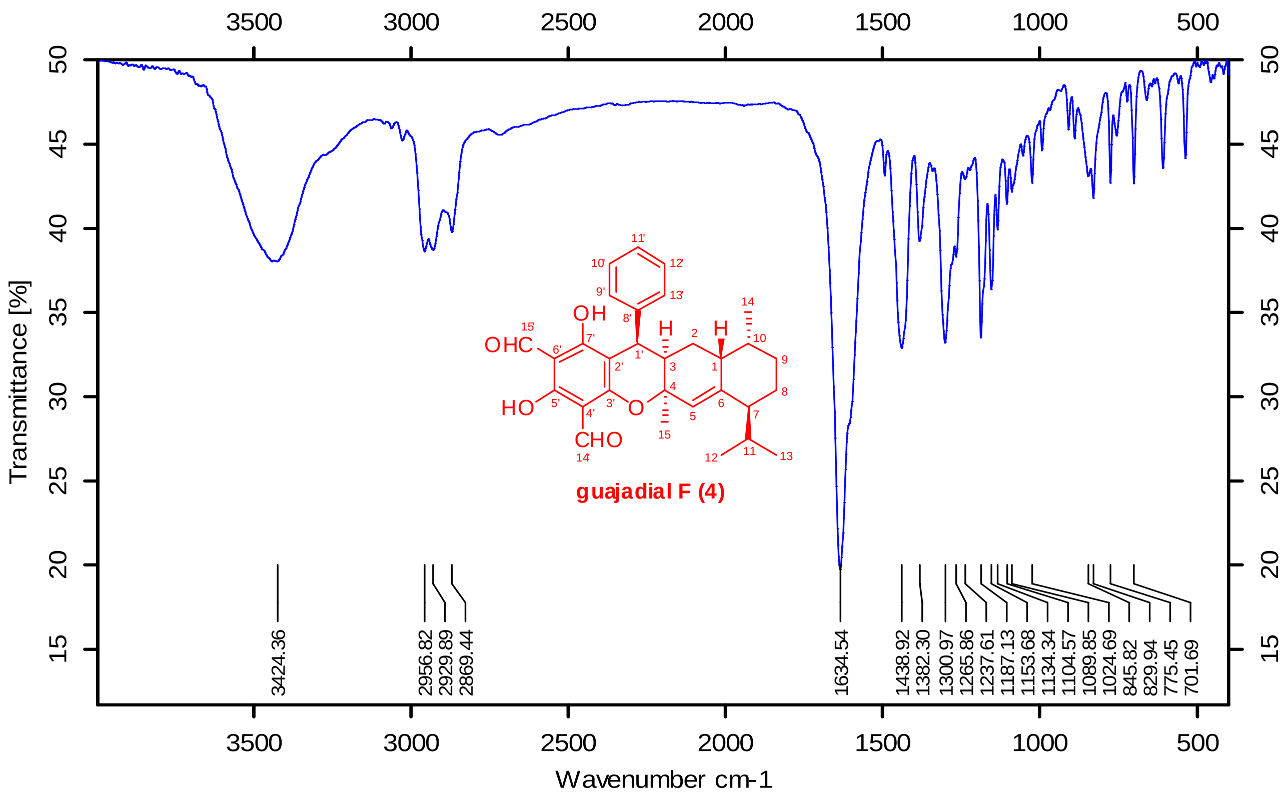


SAMPLE SCANS : 16
DATE : 31/12/2002
TIME : 17:40:36.150 (GMT+1)

SAMPLE : e2777
TECHNIQUE : KBr压片
USER : 120808IR3
